# Challenges and Perspectives on Photocatalytic Membrane Reactors for Volatile Organic Compounds Degradation and Nitrogen Oxides Treatment

**DOI:** 10.1002/gch2.202500035

**Published:** 2025-04-17

**Authors:** Muhammad Hassnain, Asad Ali, Muhammad Rizwan Azhar, Abdulrahman Abutaleb, Muhammad Mubashir

**Affiliations:** ^1^ School of Engineering Edith Cowan University (ECU) 270 Joondalup Dr Joondalup WA 6027 Australia; ^2^ Water Technologies Innovation Institute & Research Advancement Saline Water Conversion Corporation Saudi Water Authority WTIIRA‐SWA Jubail 35417 Saudi Arabia

**Keywords:** air treatment, NO*_x_* treatment, photocatalytic membrane, photocatalytic membrane reactor, VOC degradation

## Abstract

Air pollution is a pressing environmental and public health issue, with volatile organic compounds (VOCs) and nitrogen oxides (NO*_x_*) being among the most hazardous airborne pollutants. Photocatalytic membrane reactors (PMRs) have emerged as a promising technology for air purification due to their ability to integrate photocatalytic degradation and membrane separation in a single system. This paper provides a comprehensive review of the advancements, challenges, and future prospects of PMR technology for VOC degradation and NO*_x_* treatment. Various photocatalytic membranes and their fabrication techniques, including material selection, structural modifications, and catalyst immobilization strategies, are critically analyzed. The study further explores different PMR configurations, operational parameters, and their efficiency in air treatment applications. A theoretical PMR test system is also presented to evaluate design optimization strategies. Despite its potential, challenges such as membrane fouling, catalyst deactivation, and scale‐up limitations remain critical barriers to widespread adoption. Future trends focus on enhancing photocatalytic performance, developing cost‐effective materials, and optimizing reactor designs to facilitate large‐scale industrial applications of PMRs.

## Graphical abstract

Photocatalytic membrane reactors (PMRs) find the synergy of membranes as flexible, stable, permeable hosts to incorporate photocatalytic materials. This combination has strong potential in air purification systems, particularly the decontamination of air via reactive‐species‐induced degradation of volatile organic compounds and nitrogen oxides. This study critically analyzes PMR's configurational and operational parameters, along with a theoretical PMR test system.

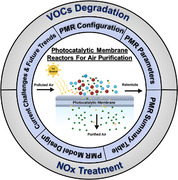

## 1 Introduction

**Figure 1:**
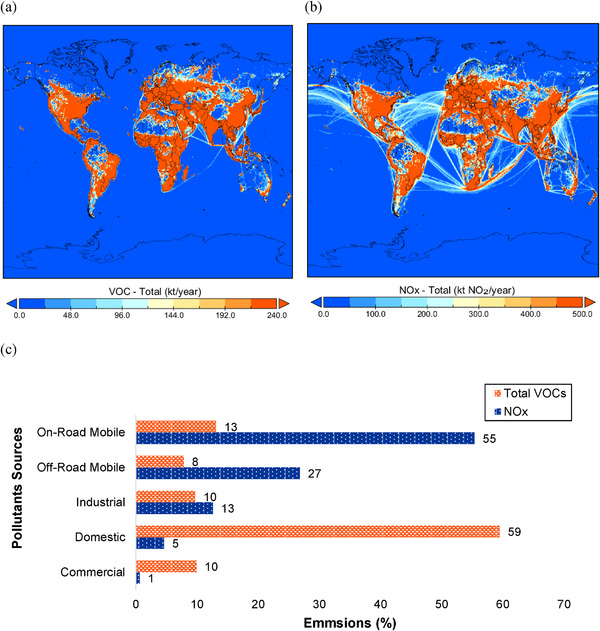
Projected countrywide emissions in 2025 (in kilotons per year) for a) total VOCs and b) NO*_x_* (represented as NO₂). Reproduced under the terms of the CC BY 4.0 License.^[^ ^15^ ^]^ Copyright 2025, Published by National Aeronautics and Space Administration. Data plotted with opensource Java Libraries in Panoply.^[^ ^16^ ^]^ c) Major sources of VOCs and NO*_x_* emissions per year in the Sydney Region, Australia. Reproduced under the terms of the CC BY 4.0 License.^[^ ^17^ ^]^ Copyright 2021, Published by Australian Government Department of the Environment and Energy.

Air pollution poses a significant threat to all life forms on Earth, primarily due to rapid population growth, urbanization, and the rapid industrial revolution occurring worldwide.^[^ ^1^ ^]^ Although air pollution may be caused by some natural phenomena, such as volcanic eruptions, windblown dust, and natural chemical emissions from organic sources, it mainly involves anthropogenic exploitation, including vast volumes of emissions from fossil‐fuel‐based vehicles, rapid deforestation, and synthetic chemical emissions from various indoor and outdoor sources.^[^ ^2^, ^3^, ^4^, ^5^ ^]^ The 2024 World Health Organization world health statistics report shows that 6.7 million people have lost their lives due to air pollution (≈4.2 million demises with climate air pollution (outdoor contamination), with ≈3.2 million deaths by household air pollution (indoor contamination)) in 2019, occurring as a result of various issues such as strokes, heart and chronic‐obstructive‐pulmonary‐based diseases, lung cancer, and acute respiratory toxicities.^[^ ^6^, ^7^, ^8^ ^]^ It is also worth noting that ≈99% of the world's population lives in areas where the air quality index has already exceeded safe limits.^[^ ^8^ ^]^ The chief sources of outdoor air pollution include the burning of solid fuels, vehicles, power generation through combustion, and the burning of agricultural waste and industrial gases.^[^ ^9^ ^]^ Indoor pollution sources primarily comprise burning fuels such as dung, wood, and coal in incompetent stoves or open hearths.^[^ ^10^, ^11^, ^12^ ^]^ Both outdoor and indoor pollution sources produce numerous health‐damaging contaminants, the most common of which are volatile organic compounds (VOCs) and nitrogen oxides (NO*_x_*).^[^ ^12^, ^13^, ^14^ ^]^ **Figure** **1** shows the global distribution of total annual emissions for VOCs and NO*_x_*, both measured in kilotons per year. The maps reveal that emissions are strongly correlated with regions of high population density and industrial activity, such as North America, Europe, South and East Asia. In Figure 1a, VOC emissions are mainly concentrated across urban and agricultural zones, reflecting contributions from both anthropogenic and biogenic sources. Figure 1b highlights NO*_x_* emissions with notable intensities over major cities, transportation corridors, and maritime shipping routes, as evidenced by the prominent pathways over oceans. The color gradient from blue to red indicates an increase in emission intensity, with red representing the highest emission levels.

In Figure 1c, most NO*_x_* emissions originate from road transport and can, therefore, be considered a major outdoor pollutant. By contrast, VOCs can be considered major indoor pollutants as the majority of these are emitted from indoor fuel burning. These contaminants are the primary cause of damage to the air quality index and major pulmonary and other diseases mentioned above. Thus, it becomes essential that utmost efforts are directed toward studies on removing these pollutants.

A variety of elimination methods are employed to address the problems, including catalytic oxidation, regenerative thermal oxidation (RTO), biodegradation, membrane filtration, photocatalytic oxidation (PCO), the use of high‐efficiency particulate air (HEPA) filters, and hybrid technologies (plasma, O_3_, and/or a combination of the former technologies).^[^ ^18^, ^19^, ^20^, ^21^, ^22^ ^]^ Among the various conventional methods, the most commonly used method is adsorption, which utilizes activated carbons or highly porous materials to remove significant pollutants from the air.^[^ ^23^ ^]^ However, these materials are limited, with issues related to regeneration and compromised adsorption, particularly in wet conditions and at lower concentrations.^[^ ^23^, ^24^ ^]^ Meanwhile, RTO technology is efficient in terms of degradation. However, this is also energy‐intensive, as biodegradation requires large‐scale facilities and is impeded by ecological variables.^[^ ^25^ ^]^ Although photocatalytic‐assisted HEPA filters can be a good fit for commercial‐scale applications, this technology is based on the selective separation of gases, which is challenging when compared to membrane‐based systems.^[^ ^26^ ^]^ Therefore, developing enhanced and novel air treatment technologies is required.

**Figure 2:**
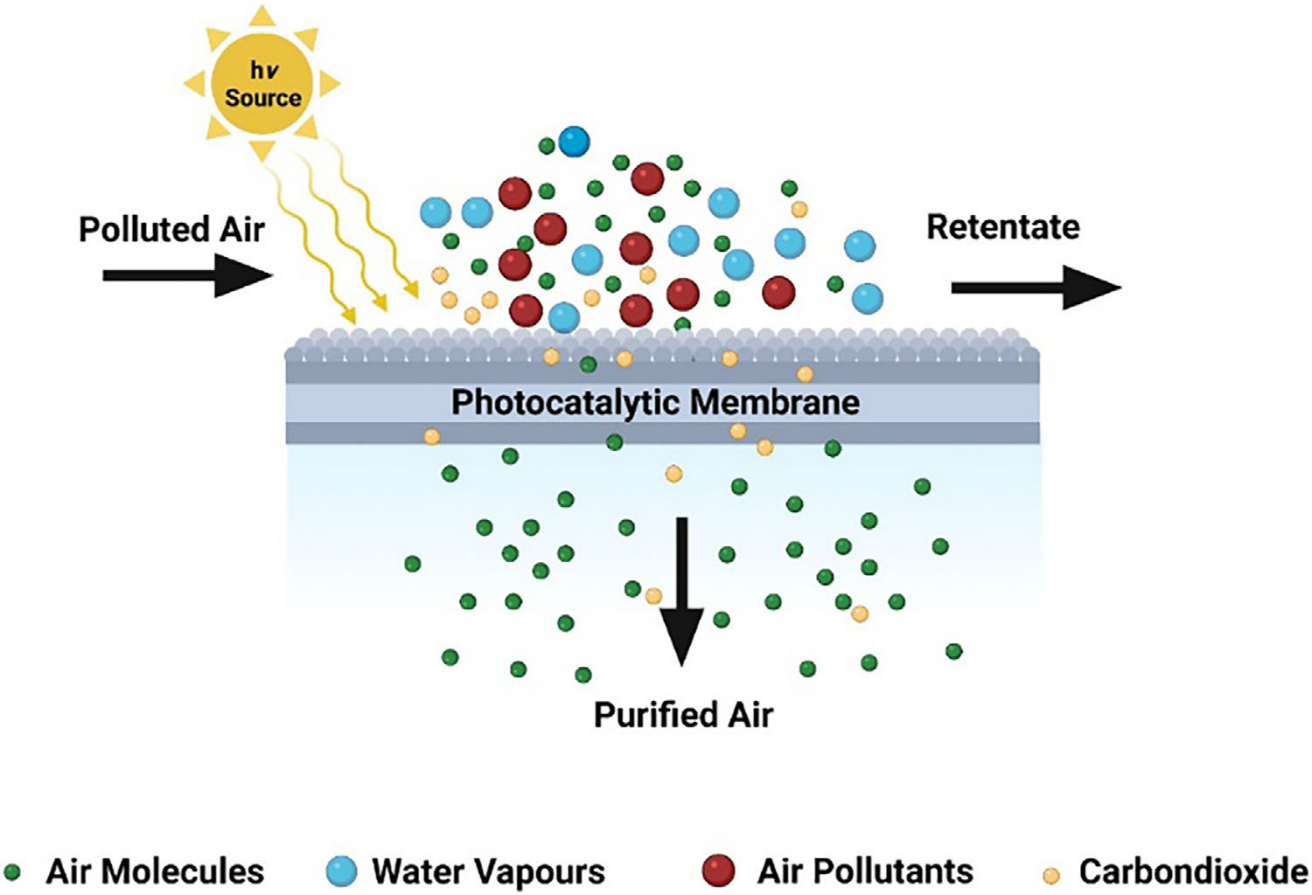
Overview of photocatalytic membrane (PM) air purification process.

Owing to its outstanding characteristics, such as ease of operation at room temperature and atmospheric pressure, effectiveness against various contaminants at low concentrations (ppb level); and less harmful final products (carbon dioxide (CO_2_) and water (H_2_O)), PCO technology appears as an alternate and encouraging technology for conventional approaches in recent years.^[^ ^27^ ^]^ During the past few years, numerous studies have been published on the development of PCO technology for air treatment.^[^ ^28^, ^29^ ^]^ Within this technology, air pollutants are not accumulated and removed from the air stream; instead, semiconductor catalysts are utilized under ultraviolet (UV) and visible and near‐infrared radiation to transform hazardous chemicals into less hazardous products.^[^ ^29^ ^]^ This is achieved by subjecting pollutants to a light source and photocatalyst, which causes the contaminants to mineralize into CO_2_ and H_2_O. A photocatalyst is a substance that absorbs light (*hv*) with an energy greater than or equal to the band gap energy (*E*_g_). It then utilizes that energy to excite electrons from the valence band to the conduction band, initiating a chemical reaction by supplying reactive species (OH• and O2^−^•) to the reactants and converting them into products, as shown in **Figure** **2**.^[^ ^27^, ^29^ ^]^

The photocatalytic process offers several advantages, such as mild operating conditions and the utilization of light and oxygen sources from the air for the degradation of various compounds in the gaseous phase.^[^ ^27^ ^]^ However, there are also numerous challenges associated with it, such as the ongoing development of high‐activity photocatalytic reactor (PR) systems. Additionally, mass transmission course and squat quantum effectiveness are the main factors that are underperforming in relation to the reaction mechanism and PR configurations.^[^ ^30^ ^]^ Additionally, redox reactions occurring in the PR may lead to the formation of harmful intermediates in cases of incomplete reactant conversions.^[^ ^31^ ^]^ These limitations often reduce the implementation of photocatalytic technologies at the industrial level.

**Figure 3:**
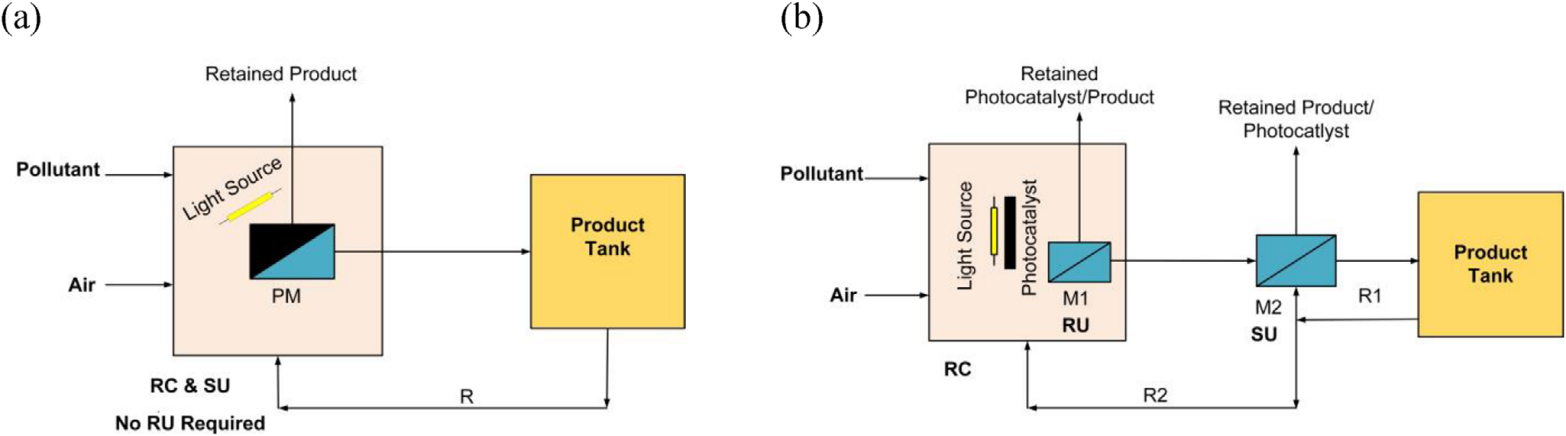
PMR arrangements as a) photocatalyst immobilized on membrane module/self‐standing membrane. b) Suspended photocatalyst with membrane inside (M1) and outside (M2) of RC.

To utilize photocatalysis for industrial applications, it is crucial to regulate chemical reaction kinetics to prevent the formation of undesirable products from secondary reaction pathways, as this leads to a low yield.^[^ ^32^, ^33^ ^]^ One of the significant disadvantages when developing PR configurations for commercial purposes is the recovery of the photocatalyst and the separation of the products.^[^ ^34^, ^35^ ^]^ Accordingly, photocatalytic membrane reactor (PMR) technology offers a possible solution in which the recovery and reuse of photocatalysts and the separation and recovery of products occur instantaneously.^[^ ^36^, ^37^, ^38^ ^]^ According to the International Union of Pure and Applied Chemistry (IUPAC) definition of membrane reactors, PMR can be distinguished as a system that operates in several arrangements and collectively couples a photocatalyst reaction with membrane separation in a single unit.^[^ ^39^ ^]^ The effective utilization of PMR‐based technology demonstrates an emergent strategy for large‐scale and industrial photocatalytic applications. The two most well‐known operative configurations are an immobilized/coated photocatalyst on a membrane network or a self‐standing membrane and a suspended photocatalyst in the reaction chamber (RC) with additional membranes (M1 and M2), as shown in **Figure** **3**.^[^ ^40^, ^41^ ^]^ Both these configurations have explicit advantages and limitations, depending on the specific applications.^[^ ^42^, ^43^ ^]^ The immobilized unit, for instance, shows synergistic effects with support. Additionally, the unit facilitates the recovery of the photocatalyst without the need for a photocatalyst retention unit (RU), enabling continuous flow operation, which is particularly beneficial for industrial applications, as illustrated in Figure 3a.^[^ ^44^ ^]^ On the one hand, the photocatalyst in the supported systems has limited access to light, leading to lower degradation efficiency and prolonged reaction times.^[^ ^45^ ^]^ On the other hand, in suspended arrangements, as shown in Figure 3b, due to their large effective area and better light harvesting, photocatalysts appear to be more photoeffective than coated photocatalysts.^[^ ^46^, ^47^ ^]^ However, the difficulty of separating photocatalysts from the reactants at the outlet stream, combined with the lack of photocatalyst suspension in a gaseous system, makes this approach less suitable for industrial gas‐phase applications.^[^ ^48^ ^]^ Thus, considering the recovery and separation of photocatalysts and the selective separation of products, both photocatalytic arrangements should be considered to achieve an effective PMR system. Due to the ease of their manufacture, coated or self‐standing PMs are primarily used for lab‐scale testing purposes and, thus, have been mainly employed in this review.^[^ ^49^ ^]^

Beyond air purification, PMRs demonstrate higher efficiency in water and wastewater treatment compared to air purification due to better mass transfer, higher pollutant concentrations, and improved photocatalyst stability in liquid‐phase systems.^[^ ^50^ ^]^ PMRs effectively degrade pharmaceuticals, pesticides, PFAS, dyes, and heavy metals in water treatment, benefiting from direct pollutant–membrane contact and photocatalyst retention, reducing leaching and deactivation.^[^ ^51^, ^52^ ^]^ Additionally, self‐cleaning properties help prevent membrane fouling, enhancing long‐term performance. By contrast, air treatment faces challenges such as low gas‐phase adsorption, slow contaminant diffusion, and catalyst deactivation due to dry conditions and exposure to UV radiation.^[^ ^53^ ^]^ Despite these challenges, PMRs remain crucial for air purification, and ongoing advancements in photocatalyst immobilization and reactor design aim to improve their efficiency for VOCs and NO*_x_* removal. While PMRs have been extensively studied for water and wastewater treatment, this study focuses solely on their application in air purification.

## 2 Photocatalytic Membranes

PMs have received attention in recent years due to their novel design, effective photocatalytic efficiency, ambient temperature applications, low energy consumption, and sustainability, as well as their synergetic photocatalytic and separation system.^[^ ^54^, ^55^ ^]^ A photocatalytic membrane usually integrates photocatalysts and separation membranes, which is advantageous for resolving the difficulties encountered in both separation and photocatalysis. The coated PM consists of two major key configurations: i) membrane with photoactive separation layer coated on the porous nonphotoactive support, and ii) nonphotoactive separation layer coated on porous photoactive support with membrane.^[^ ^43^, ^56^ ^]^ In this approach, a light source may be positioned on any side of the PM/feed lateral, depending on the type of membrane employed. Photocatalytic membrane technology has rapidly transitioned from the academic to the commercial realm, as it has surpassed state‐of‐the‐art photocatalytic performance and enabled novel functionalities, including high permeability, photocatalytic activity, and appreciable resistance to fouling.^[^ ^57^ ^]^ Modified photocatalysts are required for this technology to deliver competent photocatalytic behaviors, such as the upright departure of photogenerated charge carriers, extensive light absorption, higher durability, and reusability.^[^ ^58^, ^59^ ^]^ In PM design, key factors include loading of photocatalysts and surface modifications, mechanical strength, chemical resistance, permeability, selectivity, and the structure/morphology of the membrane.^[^ ^35^, ^60^ ^]^ These parameters need to be optimized to achieve outstanding activity of PMR in various air and water treatment applications.^[^ ^61^ ^]^

**Figure 4:**
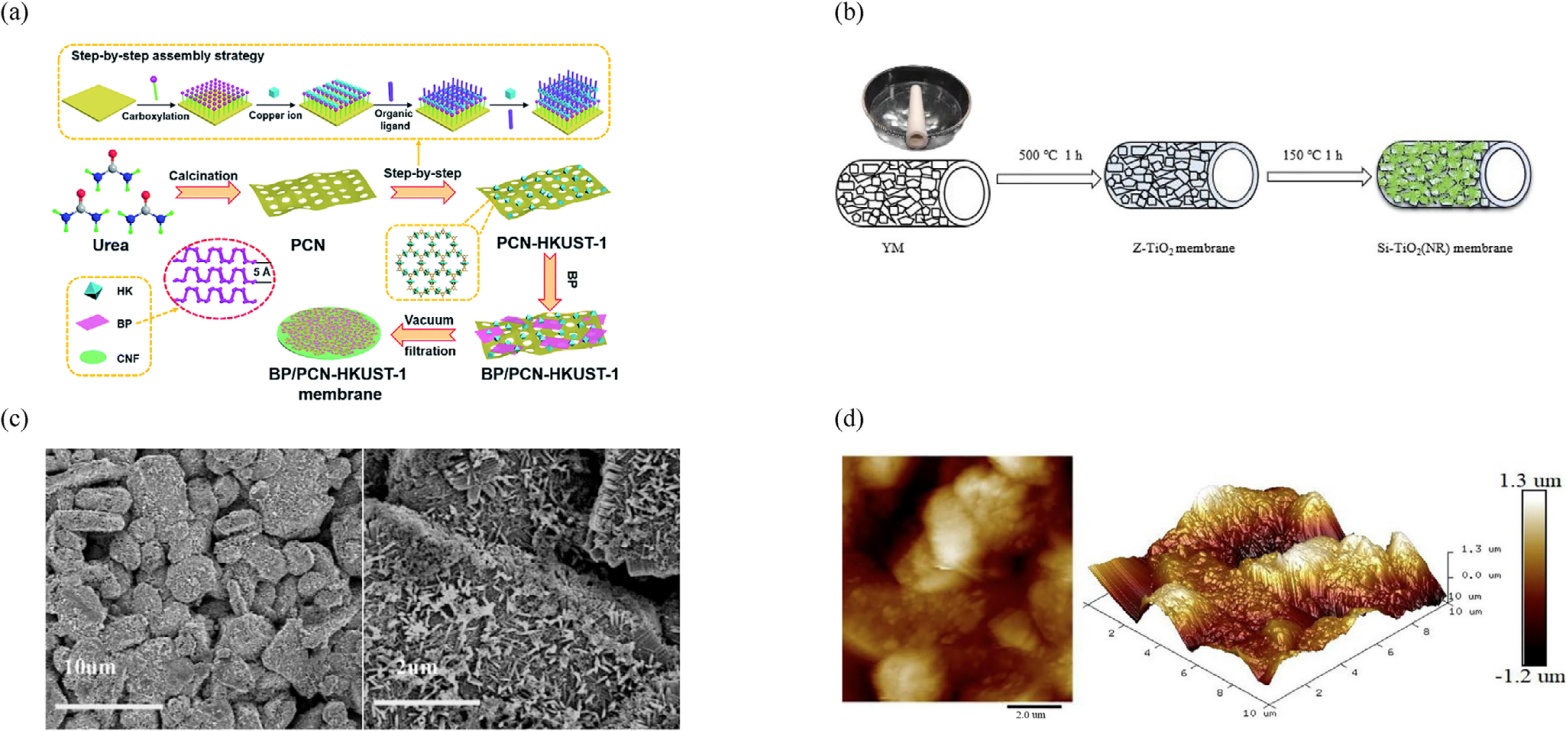
PM preparation steps for a) BP/PCN–HKUST‐1 membrane. Reproduced with permission.^[^ ^64^ ^]^ Copyright 2019, The Royal Society of Chemistry. b) Silicon‐doped TiO_2_ nanorod (Si─TiO_2_ NR) alumina hollow ceramic membrane with characterization techniques as c) SEM of Si─TiO_2_ NR membrane (top surface) and d) AFM 2D (left)/3D(right) image (top surface). Reprodued with permission.^[^ ^46^ ^]^ Copyright 2014, Elsevier.

The choice of suitable materials from a variety of organic, inorganic, and/or metallic moieties, along with immobilization strategies (dip coating, tape casting, dry–wet spinning, drop‐casting, electrospraying, electrospinning, vacuum filtration, and so on), are summarized in **Figure** **4** for the novel scheme, which is established for photocatalytic membranes involving numerous gas treatment applications under UV and UV–visible light irradiation.^[^ ^62^, ^63^ ^]^ For example, Hu et al. have detailed the synthesis of black phosphorus (BP)/polymeric carbon nitride (PCN)–HKUST‐1 membranes, as shown in Figure 4a.^[^ ^64^ ^]^ The figure indicates that the photocatalyst is immobilized on the surface of the membrane through a series of steps, utilizing the vacuum filtration method. PCN demonstrated a highly porous and thin nanosheet morphological structure, whereas BP exhibited ultrathin and smooth nanoflake structures. In addition to the preparation method itself, the physicochemical characteristics of the photocatalyst and the surfaces of the synthesized membrane play a crucial role in PM preparation.^[^ ^58^, ^65^ ^]^ Additionally, critical issues in the choice of membrane and photocatalyst include photochemical durability, adhesion of the photocatalyst to the membrane module, enhancement of quantum efficiency, type of light spectrum, and mechanical strength.^[^ ^40^, ^66^ ^]^ In terms of permeability and selectivity, membrane transport properties must also be measured. These can be tailored using a suitable membrane structure and configuration selection.^[^ ^67^ ^]^ Additionally, photocatalysts must be firmly embedded or supported on the membrane support for optimal performance. In general, mechanically durable support/ceramic materials such as alumina, silica, zirconia, titania, and metal/oxide mixtures have been employed as photocatalytic membrane materials.^[^ ^68^, ^69^ ^]^ These ceramic membranes are primarily fabricated through the sol–gel technique, where dispersions are used to prevent the formation of aggregates.^[^ ^48^ ^]^ Accordingly, asymmetric assembly is achieved by dropping particles of decreasing size and calcination at elevated temperatures to form continuous, porous layers. The same method is employed, as shown in Figure 4b, to prepare an alumina hollow ceramic membrane using Si–titanium dioxide (TiO_2_) nanorods (NRs). Interestingly, the pore dimensions and features of the superior selective region can be adjusted according to the grain size and type of particles used.^[^ ^46^ ^]^ Figure 4c,d presents the surface characterization of this membrane as scanning electron microscopy (SEM) and atomic force microscopy (AFM) for surface morphological and topographical (2D/3D) analysis, respectively.^[^ ^70^ ^]^

**Figure 5:**
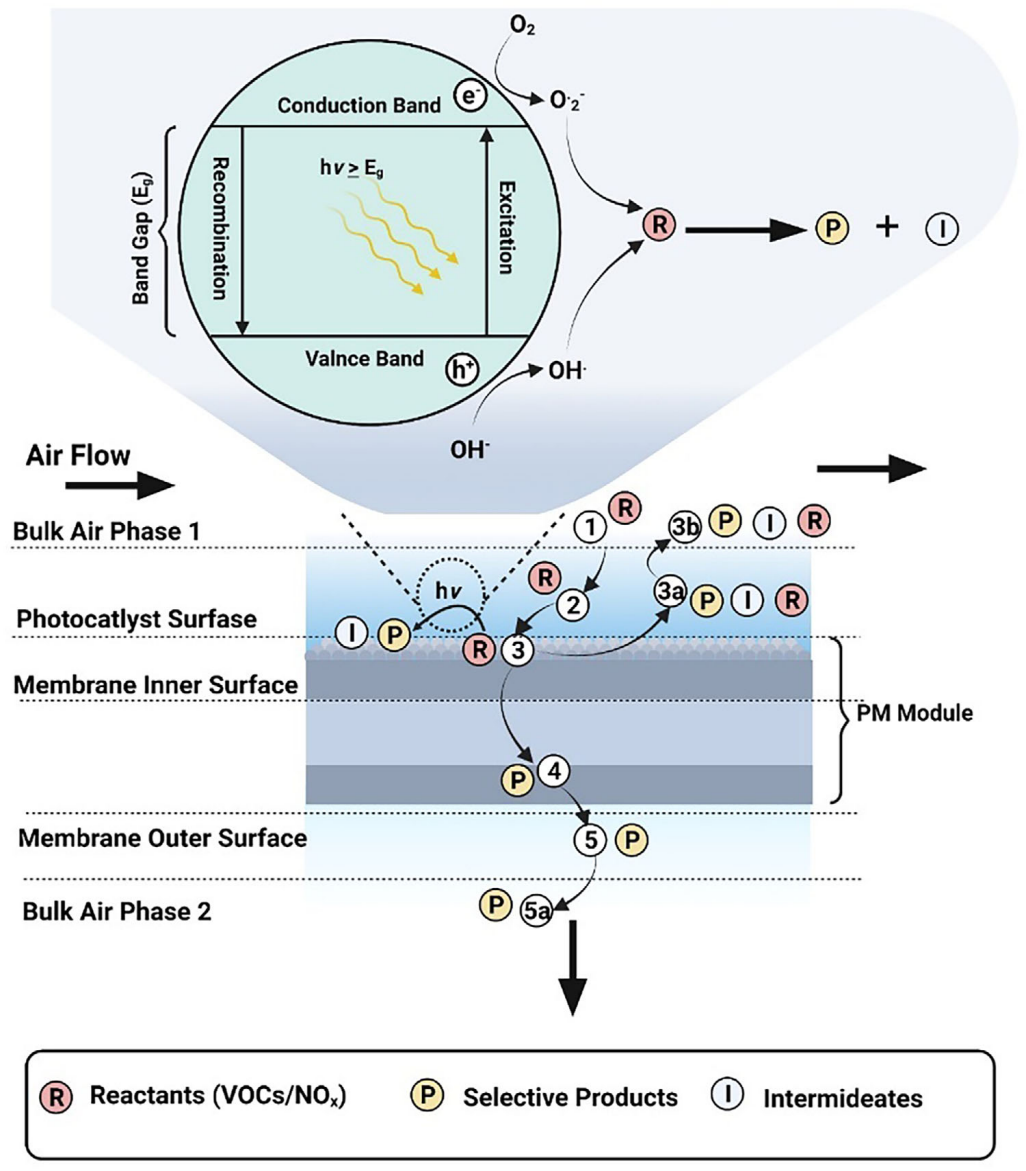
General photocatalytic reaction and transportation mechanism through photocatalytic membrane.

In terms of reaction mechanism, PM follows the step‐by‐step complexity of both external and internal mass transfer, as shown in **Figure** **5**.^[^ ^71^ ^]^ External reactant particle mass transfer from the bulk phase to the PM surface occurs in Steps 1 and 2. P and I are produced in Step 3 in the presence of light and transported to the surface (3a–3b) or membrane matrix (3–4) as retentate for separation. Finally, the permeate bulk side selective product (5–5a) is obtained. In addition to the reported literature on these steps, it can be assumed that the complete separation of products (P) occurs with some reactants (R) that are also converted into intermediates (I).

Various studies have reported ceramic and polymeric PM for air treatment applications (including NO*_x_* and VOC treatment).^[^ ^72^, ^73^, ^74^ ^]^ Ceramic membranes, such as TiO_2_, zinc oxide (ZnO), ferric oxide, cadmium sulfide (CdS), gallium phosphide, zinc sulfide, and others, have been employed as stable photocatalysts and substrates.^[^ ^75^, ^76^ ^]^ Pure freestanding TiO_2_ PMs are also commonly used and are developed mainly by utilizing TiO_2_ nanomaterial.^[^ ^77^ ^]^ Various TiO_2_‐based PMs have been assessed by distinctly describing membrane applications or quantifying photocatalytic activities.^[^ ^77^, ^78^, ^79^ ^]^ Moreover, research has indicated that the photocatalytic performance of TiO_2_‐based materials can be enhanced using various approaches, such as loading metal particles (platinum (Pt), Au, silver (Ag), Pd, and so on), doping of nonmetallic elements (C, N, P, and S) and hosting heterojunctions or immobilizing on carbon materials.^[^ ^80^ ^]^ For instance, Modesti et al. have developed three different TiO_2_‐based membrane nanomaterials in this regard: TiO_2_ nanofibers, polymer TiO_2_ nanofibers, and multilayer polymer nanofibers with TiO_2_ electrospray derived for the photooxidation of VOC (methanol).^[^ ^81^ ^]^ 2D graphitic carbon nitride (g‐C_3_N_4_) nanosheets have gained substantial attention as a novel photocatalyst material due to their large surface area, exceptional visible light activity, tunable surface functions, and ease of structural modification through the addition of different serving materials.^[^ ^82^, ^83^, ^84^, ^85^ ^]^ Several research studies have also been made to enhance the surface properties of the membrane, including elemental doping, chemical modification, and building of heterojunctions (CdS/g‐C_3_N_4_, PB/g‐C_3_N_4_, NiS_2_/g‐C_3_N_4_, WS_2_/g‐C_3_N_4_, molybdenum disulfide (MoS_2_)/g‐C_3_N_4_, CoTiO_3_/g‐C_3_N_4_, tungsten oxide (WO_3_)/g‐C_3_N_4_, g‐C_3_N_4_/reduced graphene oxide (rGO), and so on) for numerous photocatalytic applications.^[^ ^86^, ^87^ ^]^ Recently, Hu et al. have reported on BP embedded in a porous g‐C_3_N_4_(PCN)/metal–organic framework (MOF) heterojunction as a novel photocatalytic membrane material for removing nitric oxide from air under visible light radiation.^[^ ^64^ ^]^

Several polymers, such as polyamide (PA), polyvinylidene fluoride (PVDF), polyethersulfone (PES), PVDF/sulfonated polyethersulfone (SPES), polyurethane (PU), polyethyleneterephthalate (PET), polyester, polyacrylonitrile (PAN), and polytetrafluoroethylene (PTFE) have also been employed as supports in photocatalytic membranes.^[^ ^35^, ^88^ ^]^ The choice of polymeric materials for PMRs is crucial as these materials must provide the necessary stability, permeability, mechanical strength, and compatibility with photocatalysts.^[^ ^89^ ^]^ PA is known for its excellent mechanical strength, chemical resistance, and hydrophilicity; PA membranes are widely used in filtration and separation processes. Their high surface charge enhances interaction with photocatalysts, improving pollutant degradation efficiency.^[^ ^90^ ^]^ PVDF membranes exhibit high thermal and chemical stability, making them suitable for harsh operating environments. They have excellent hydrophobicity, which can be modified through surface treatments to enhance photocatalyst adhesion and water permeability.^[^ ^91^ ^]^ PES is known for its high permeability, mechanical durability, and oxidation resistance.^[^ ^92^ ^]^ SPES, a modified form of PES, features additional hydrophilic functional groups that enhance flux and photocatalyst dispersion, thereby improving photocatalytic activity.^[^ ^93^ ^]^ PU membranes are highly flexible and resistant to mechanical stress, making them suitable for applications that require both membrane elasticity and durability. Their structure allows for the effective immobilization of photocatalysts while maintaining membrane integrity.^[^ ^94^ ^]^ PET and polyester are polymers that provide high chemical resistance and thermal stability. Their smooth surface morphology facilitates the controlled deposition of photocatalysts, reducing fouling and enhancing long‐term performance.^[^ ^95^ ^]^ PAN membranes have high porosity, chemical stability, and good mechanical strength. They are widely used in gas‐phase and liquid‐phase photocatalysis, providing a stable support for heterogeneous photocatalysts.^[^ ^96^ ^]^ PTFE is known for its exceptional resistance to extreme pH conditions, thermal stability, and nonstick properties. It is ideal for harsh chemical environments and can be modified to improve photocatalytic performance.^[^ ^97^ ^]^

Accordingly, the PMs were shown to possess upright thermal durability, extraordinary energy efficiency, and low cost, making them suitable for various industrial applications. Covalent organic frameworks have also been established as a growing area of interest for gas conversion, storage, and separation applications, owing to their high porosity with chemical functionalities, thermal durability, and a large volume of proton conductivity.^[^ ^98^ ^]^ Hence, the effective employment of PM features is a fundamental tactic to expand a beneficial blend of photocatalytic processes and membrane technology concurrently. Through single‐unit photocatalytic reactions and product separation, synergetic effects enhance both quantum and economic impact.

## 3 Photocatalytic Membrane Reactor

In recent years, PMR has emerged as a promising technology for overcoming the disadvantages of conventional reaction separation techniques, as well as the limitations of photocatalysis alone.^[^ ^35^, ^99^ ^]^ PMR, or membrane photocatalytic reactor, is an integrated system consisting of photocatalytic reaction and membrane filtration processes into a single unit. This system offers many key advantages such as i) low capital/operating cost, where the design and installation of PMR are cost‐effective and easy, the reaction/separation integrated PMR module constitutes lower overall costs, the system needs comparatively small reaction volume, it has a lower cost of preparation of the membrane, and membrane generally needs a small amount of material to coat/embed; ii) operation at ambient conditions: mainly PMR operation needs ambient conditions as the use of light triggers the reactions;^[^ ^100^ ^]^ iii) ease and trustworthiness: the membrane‐based materials remain stable in the long run, as they do not exhibit rapid decay in routine and thus avoid regular shutdown and start‐up;^[^ ^43^ ^]^ iv) self‐cleaning ability and reduced membrane fouling: impurities on the surface of the PM are constantly degraded due to photocatalytic surface reactions, creating a self‐cleaning effect that leads to reduced fouling (fouling is a process of adsorption and/or deposition of materials on the pore and surface of the membranes, developing flux decrement, PM erosion/rupture/channeling, and even decrease membrane durability);^[^ ^100^, ^101^ ^]^ v) adaptability and design efficiency: PMR functions to eliminate a significant percentage of pollutant gases and help separate residue products, with the ability to confine photocatalyst within the reactor.^[^ ^102^ ^]^

Due to several advantages, PMR has been employed in numerous applications, including air treatment processes such as the removal of VOCs and NO*_x_*. A detailed description of PMRs is provided in Sections 3.1.1 and 3.1.2, analyzing both operational parameters and outputs. **Table** **1** provides a summary of PMRs for VOC degradation and NO*_x_* treatment with PM employed, as well as the reactor configuration and operational conditions required for the development of photoactive materials with desirable characteristics necessary for the efficient design of PMR systems. Moreover, numerous systematic investigations in these areas have been conducted on a pilot scale, with varying operating conditions, to evaluate the PMR methodology.

**Table 1:** Summary of PMs and PMRs for VOC degradation and NO*_x_* treatment. Abbreviations: polyvinylidene fluoride (PVDF), graphene oxide (GO), graphene (G), polyacrylonitrile (PAN), α‐alumina (α‐Al_2_O_3_), diameter (*D*), titanium silicate (TS‐1), hybrid silicate (Sil‐1), stainless steel (SS), 1‐hexyl‐3‐methylimidazolium hexafluorophosphate ([HMIm]PF_6_), neodymium─TiO_2_ (Nd─TiO_2_), Aquivion D83‐06A (AQ), Hyflon AD60 (AD), supported ionic liquid membrane (SILM), reduced‐GO TiO_2_ composite (rGO─TiO_2_), polydimethylsiloxane (PDMS), water (H_2_O), tungsten oxide (WO_3_), hydrogen peroxide (H_2_O_2_), polypropylene (PP), TiO_2_/polyimide (TiO_2_/PI), black phosphorus (BP), porous carbon nitride (PCN), copper benzene tricarboxylate porous material (HKM), nitrogen oxide (NO), molybdenum disulfide (MoS_2_), bismuth subcarbonate (BOC), carbon nanofibers (CNFs), recycled fine glass without TiO_2_ (RG‐T_0_), RG with 2% TiO_2_ (RG‐T_2_), photocatalytic materials (P1, P2), control (C1), hollow porous carbon nitride nanosphere coupled (HCNS), carbonized polymer nanofibers (CNCFs), titanate (Ti), silicon‐doped TiO_2_ nanorod (Si─TiO_2_ NR), height (*H*), Fe doping TiO_2_/polysulfone (FeTiO_2_/PSF), polyimide (PI), manganese oxide (MnO*_x_*), polymeric carbon nitride (CN), platinum (Pt), manganese oxide (MgO), polyacrylic acid (PAA), polyethersulfone (PES), metal–organic frameworks (MOFs), Materials Institute Lavoisier (MIL), silver (Ag), amino radical (NH_2_), zinc/aluminum (ZnAL), zinc oxide (ZnO), layered double hydroxide (LDH), gas mixture (GM), width (*W*), length (*L*), internal diameter (ID), volume (*V*), ultraviolet (UV), pressure change/drop (Δ*P*).

Air purification application	PMR configuration	PM module configuration			Targeted pollutant	PMR operating conditions						PMR efficiency^a)^	Refs.
		Photocatalyst/membrane/support	Photocatalyst/PM preparation method	Immobilization technique		Feed concentration/flow rate	Relative humidity conditions	Light source	Gas/air flow rate	Photocatalyst loading	Temperature/pressure		
VOC degradation	Continuous photocatalyst membrane biofilm reactor	N─TiO_2_/PP	Sol–gel method and membrane surface coating	Impregnation method	Toluene	140 mg m^−3^	–	Visible light	GM: 60 mL min^−1^	–	–	≈99%	[114]
	Batch Pyrex tubular reactor (*L*: 370 mm, ID:100 mm, *V*: 2780)	Graphene─TiO_2_/PVDF nanofibers	Solvothermal method	Electrospinning method	Methanol and acetaldehyde	Methanol: 530 ± 40 ppm acetaldehyde: 1100 ± 150 ppm	Dry air circulation	UV light (1.35 W m^−2^)	30 L min^−1^	mg m^−2^ (× 10^3^) PVDF TiO_2_: ≈0.41 PVDF TiO_2_─G: ≈0.85 PVDFTiO_2_─GO: ≈0.82	50 °C	100%	[115]
	Lab‐made reaction device (*V*: 0.5 L)	TiO_2_/PAN	Ready‐made photocatalyst and membrane	Electros prying/electrospinning	Toluene	325 ± 50 mg m^−3^	–	Xenon light	32 L min^−1^	0, 4, 9, 13, and 22 wt% of TiO_2_	20 ± 5 °C	97.4%	[110]
	Cylindrical glass tube membrane module (with 3 pass flow pattern)	Porous TiO_2_/α‐Al_2_O_3_	Sol–gel Process	Coating colloidal sols	Methanol	500–6000 ppmv/50–500 cm^3^ s^−1^	–	UV light (4 W, 350 nm)	GM: 50–500 cm^3^ s^−1^	–	100 °C/10–50 kPa	Enhanced rate constant with membrane module (from 2.5 to 1.5 × 10^−6^ m s^−1^)	[116]
	Cylindrical glass tube membrane module (*D*: 8 × 10 mm *L*: 9 cm with 3 pass flow pattern)	Pt‐modified porous TiO_2_/α‐Al_2_O_3_	Sol–gel Process	Coating colloidal sols	Methanol and Ethanol	100 ppm	–	UV light irradiation (black light, 4 W, 350 nm)	500 × 10^−6^ m^3^ min^−1^	–	10–20 kPa	100%	[109]
	Pyrex glass covered stainless steel rectangular reactor (*L*: 578 mm, *W*: 113 mm)	TiO_2_‐based membranes a) TS‐1/SS b) Hybrid Sil‐1/SS	Modified sol–gel process	Coating method	Trichloroethylene	240 ppmv	–	UV light (6 W)	GM: 200 sscm	Fixed TiO_2_ content of 0.2 mg for TS‐1/SS membrane	Δ*P*: 1 Psig	52%	[117]
	Batch reactor as black chamber with Teflon film coating (*V*: 0.5 m^3^)	[HMIm]PF_6_/Nd─TiO_2_ SILM)	Sol–gel method	Spreading	Toluene, acetone, benzene, xylene, and chloroform	1000 ppm which can be reduced to 200–400 ppm for 10 h operation	–	UV–vis light (500 W cutoff <420 nm or >420 nm)	Sweep air: 0.5 L min^−1^	Nd content in Nd─TiO_2_ (wt%):0.5, 0.8, 1, and 1.5	10 kPa (vacuum) on permeate side	60–80%	[108]
	Pyrex tubular batch reactor with 5 membrane modules (*L*: 370 mm, ID: 100 mm, *V*: 2.78 L)	TiO_2_‐based membranes a) TiO_2_ nanofibers b) Nanocomposite membranes c) Multilayer membranes	Electrospinning, electrospraying, and pyrolysis	Electrospinning, electrospraying, and pyrolysis	Methanol	Methanol in air stream: 1350 ppm	Dry air saturated with VOC	UV light (16 W, 250 nm)	GM: 155 cm^3^ s^−1^	TiO_2_ content (mg cm^−2^) type 1 (4.25), type 2 (2.13), and type 3 (0.69, 1.2, and 2.68)	25 °C	Batch mode type 1: 80%, type 2: 40%, type 3: 100%	[81]
	Pyrex tubular batch reactor with 5 membrane modules (*L*: 370 mm, ID: 100 mm, *V*: 2.78 L)	Graphene/TiO_2_‐based membranes type A (TiO_2_), B (G/TiO_2_), and C (rGO/TiO_2_ composite)	Electrospraying and hydrothermal treatment	Electrospraying	Methanol	Methanol in air stream: 3100 ppm	Dry air saturated with VOC	UV light	GM: 155 cm^3^ s^−1^	TiO_2_ content (mg cm^−2^) A (≈0.6), B (≈0.37), and C (≈0.55)	–	100%	[137]
	Pyrex batch glass reactor equipped with stirrer and quartz window cover for irradiation (*V*: 145 mL)	TiO_2_/AD–AQ membrane	Premade photocatalyst embedded into membrane matrix	Coating method (multilayered deposition of perfluoro polymers on quartz window)	Dichloromethane, methanol, 2‐propanol, pentane, pyridine, and toluene	2000 and 5000 ppm_v_	Dry condition: ≈ 20% wet condition: 500%	UV light (21 mW cm^−2^)	–	TiO_2_ amount on AQ content (wt %) 10%	85 and 20 °C.	High photodegradation rate of >10^−5^ s^−1^ for all VOCs	[138]
	Hybrid plug flow reactor with membrane and permeate side sweep gas flow. *V*_Permeate_/*V*_Retentate_ (mL):65/145 mL	TiO_2_/PDMS and TiO_2_/PDMS/PAN support	Sol–gel TiO_2_ with predefined properties	–	*n*‐hexane	Inlet concentrations: 1, 5, 10, and 25 ppm	Feed side relative humidity (RH): 1%	UV‐A light (310/400 nm)	GM: 50 and 100 NmL min^−1^	Catalyst content (g): 0.15, 0.41	24 °C	100%	[107]
	Cylindrical‐ laboratory scale water jacketed Pyrex glass reactor (4 L of gas treated)	a) TiO_2_/PP b) WO_3_/PP c) (TiO_2_/WO_3_)/PP	TiO_2_ graft polymerization onto PP matrix	Photografting	Methane	10–1000 ppm	100% (H_2_O)	UV light, (500/20 W, 400/254 nm)	–	TiO_2_/WO_3_ blend content (wt%): 30 ± 3	318 ± 3 K	(TiO_2_/WO_3_) blend shown highest value of *ø_∞_* at a wide range of irradiance	[118]
	Cylindrical‐ laboratory scale water jacketed Pyrex glass reactor (*V* of gas treated: 4.0 + 0.005 L)	TiO_2_/PP	TiO_2_ graft polymerization onto PP matrix	Photografting	*n*‐alkanes (methane, ethane, and *n*‐heptane)	10–1000 ppm	100% (H_2_O_2_)	UV light, (500/20 W, 400/254 nm)	–	TiO_2_/WO_3_ blend content (wt%): 30 ± 3	≈308 K	100%	[139]
	Laboratory‐made batch reactor (*V*: 1 L)	TiO_2_/PI composite membrane	Simultaneous electrospinning and calcination at 400 °C	Simultaneous electrospinning and calcination at 400 °C	Toluene	400 ± 50 mg m^−3^	–	UV light (40 W)	–	Variable TiO_2_ content based on TiO_2_/PI ratio	20 ± 5 °C	100%	[140]
	Batch reactor (0.5 L)	GO/MnO*_x_*/CN	Soft‐chemistry exfoliation	Facile filtration	Formaldehyde	160 ppm	–	UV–vis light (300 W)	–	Variable catalyst loading ratios for G–Mn–CN film	24.3 °C	>90% in 12 min of irradiation for optimal loading of 8:1 G–Mn–CN film	[119]
	Quartz cylindrical reactor stainless steel mesh coated with catalyst	Pt/TiO_2_─ZnO	Sol–gel method	Physiochemical processes	Formaldehyde	0.21 mg m^−3^	–	UV–vis light (250–800 nm)	0.00556 m^3^	0.4%, 0.6%, 0.8, 1% Pt/TiO_2_─ZnO	25 °C	74% removal in 2 h with optimum loading of 0.8%	[120]
NO*_x_* treatment	Stainless steel, quartz glass covered cylindrical continuous flow reactor (*D*: 12 cm, *L*: 20 cm)	BP/PCN–HKM	Series of physical and chemical processes	Vacuum filtration	NO	600 ppb of NO	50%	Visible light (300 W)	1.2 L min^−1^	Fixed photocatalyst amount of 150 mg	25 °C	74%	[64]
	Cylindrical, glass made continuous flow reactor (*D*: 10 × 20 cm, *V*: 1.6 L)	CNFs BOC–CNFs BOC–MoS_2_–CNFs	Electrospinning/hydrothermal method	Self‐standing membrane	NO	600 ppb	50%	Visible light (300 W)	GM: 2.4 L min^−1^	Fixed loading of 0.15 g	25 °C	68%	[141]
	Single pass continuous flow reactor with Teflon membrane filters and glass container	Nano‐TiO_2_/membrane filter a) RG‐T_0_ b) RG‐T_2_	Premade TiO_2_	Physical spreading	NO*_x_*	1000 ± 50 ppb	30%	UV‐A (340 nm, 2 W m^−2^)	3 L min^−1^	TiO_2_ content RG‐T_0_ (0%), RG‐T_2_ (2%)	25 °C	28 µmol m^−2^ h^−1^	[130]
	Reactor configuration according to ISO standard 22197‐1	Architectural membranes with TiO_2_ coating P1, P2, and control (C1)	Ready‐made membranes were used	–	NO removal NO*_x_* deposition	1000 ppb	50%	UV‐A (360 nm, 11.5± 1.5 W m^−2^)	3 L min^−1^	–	60 °C	Highest removal rate: ≈0.8–1 µmol h^−1^	[131]
	Glass made cylindrical reactor (*D*: 10 × 20 cm, *V*: 1.6 L)	(HCNS/rGO)/CNCF	Modified Hummer's method/electrostatic interaction	Electrospinning	NO	NO: 100 ppm gas mixture: 600 ppb	50%	Visible light (20 W)	2.4 L min^−1^	–	25 °C	64%	[132]
	Glass made cubic lab scale batch reactor	TiO_2_ nanoparticles/PVDF/PDMS	Electrospinning	Electrospinning	NO	–	–	UV light	–	0.5 and 1 wt%	–	100%	[133]
	Stainless steel self‐designed batch photoreactor by using simulated gas flue	TiO_2_ membrane	Hydrothermal treatment/liquid phase deposit	Self‐standing	NO	Different concentrations 628, 942, 1256 mg m^−3^ 1569 mg m^−3^	4–10%	UV light (40 W)	0.064 m^3^ h^−1^	Fixed amount	25 °C	67%	[142]
	Self‐designed stainless‐steel batch photoreactor (*H*: 1.4 m, *D*: 80 mm)	TiO_2_ membrane	Supported photocatalyst self‐prepared	Self‐standing	NO	1081 mg m^−3^	0.006 m^3^ m^−3^ of water	UV light	0.128 m^3^ h^−1^	Fixed amount	100 °C	50%	[125]
	Rectangular stainless steel continuous flow reactor (*L*: 15, *W*: 10, *H*: 10 cm)	Si─TiO_2_ NR membrane	Hydrothermal method	Seed‐coating	NO	300 ppm	70%	UV irradiance (125 W)	300 mL min^−1^	8.639 wt % of Ti	50 °C	Removal: 67.11% separation: 56.82%	[121]
	Hollow fiber hybrid catalytic membrane biofilm reactor	FeTiO_2_/PSF hybrid membrane	Sol–gel method	Impregnation method	NO	23.3–216.3 g m^−3^ h^−1^	Aqueous medium	Visible light (620–700 lux)	0.1 L min^−1^	–	25–55 °C	Removal: 85.7%	[122]
	Stainless steel with quartz window cubic continuous flow reactor (*L*: 15, *W*: 10, *H*: 10 cm)	Si─TiO_2_/Al_2_O_3_	Hydrothermal treatment	Seed‐coating	NO	50 ppm	Saturated water vapors	UV light (125 W)	120 mL min^−1^	*x*% Si─TiO_2_ with *x*% as: 0%, 0.2%, 0.4%, 0.6%, and 1%	20–120 °C	Optimum loading: 0.2% NO conversion: 100% in 11 min water vapor recovery efficiency: 7.04%	[134]
	Rectangular stainless‐steel reactor with glass window	Ag/ZnO/ZnAL‐LDH	Sol–gel method	Photoreduction	NO	500 ppb	Controlled humidity	Visible light (300 W)	–	0.025–0.2 g of ZnO on PP	–	Enhanced NO removal efficiency of 46.53% on 3% Ag/ZnO/PP membrane	[135]
	Homemade reactor (*V*: 25 L)	MOFs UiO‐66, UiO‐66─NH_2_, MIL‐125, and MIL‐125─NH_2_	UiO‐66 and UiO‐66─NH_2_: reflux solvothermal process MIL‐125 and MIL‐125─NH_2_: reflux protocol	Solution dispersion and freeze drying	NO*_x_*	500, 750, and 1000; ±50 ppb	–	UV‐A (320–400 nm, 20 W m^−2^)	–	Sorbent/catalyst ratios (10, 25, and 50 mg)	–	100% degradation under UV‐A irradiation and independent of catalyst loading and NO*_x_* initial concentration	[136]

^a)^ PMR efficiency may refer to removal, separation, or degradation efficiency.

### 3.1 Applications of PMR in Air Purification

#### 3.1.1 Photodegradation of VOCs in Air

According to the United States Environmental Protection Agency, the existence of high VOCs both indoors and outdoors often has severe adversative effects on the environment and civic health.^[^ ^103^, ^104^, ^105^ ^]^ A variety of VOCs are released following the use of a range of products, including paints, cleaning materials, pesticides, construction supplies, fittings, and others. Subsequently, among the numerous technologies involved in improving indoor and outdoor air quality, PMRs are considered the emerging technology, integrating photodegradation and membrane removal of VOCs, as discussed in previous sections.^[^ ^99^, ^100^, ^106^ ^]^

**Figure 6:**
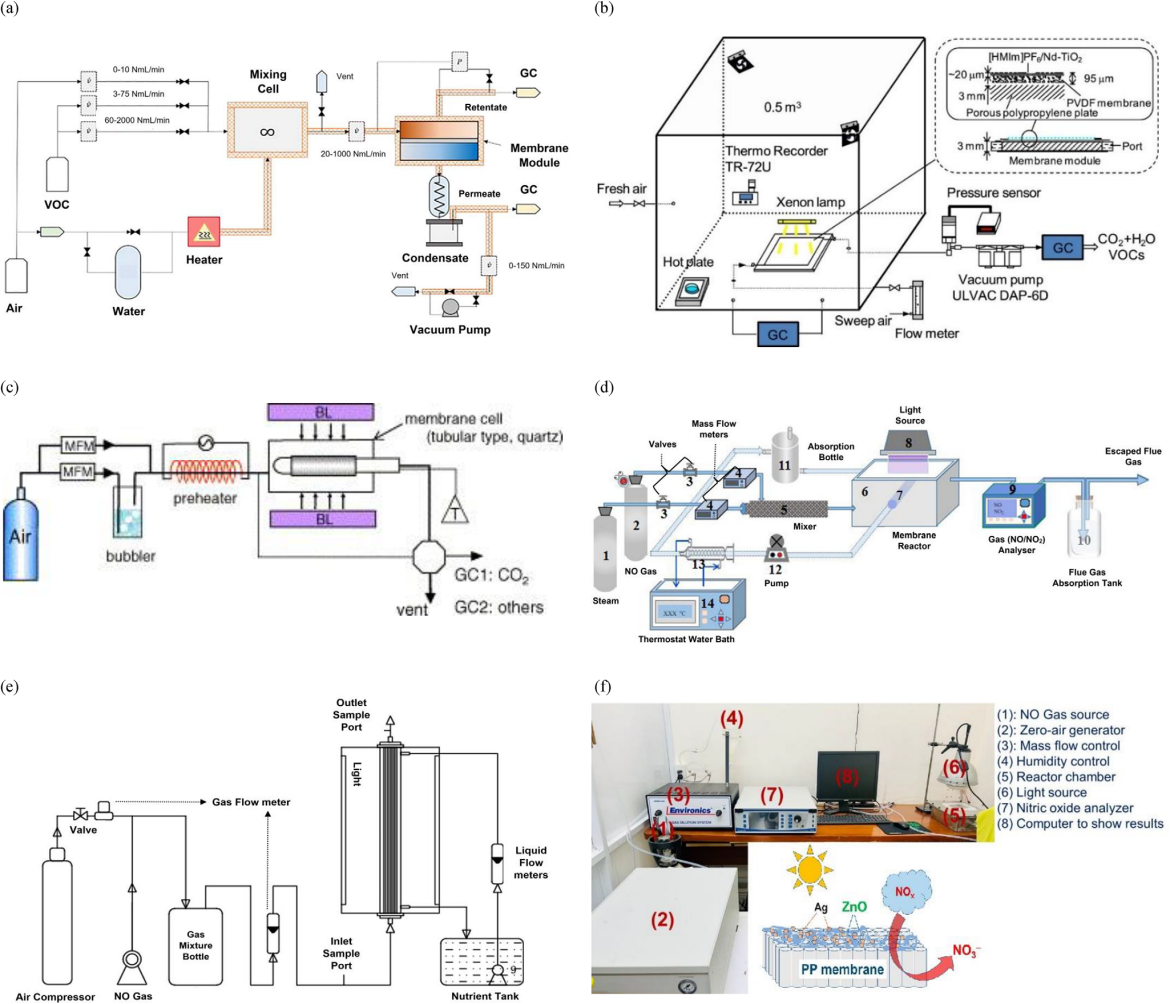
a) Schematic diagram of PMRs for the degradation/treatment of methanol. Reproduced with permission.^[^ ^107^ ^]^ Copyright 2021, Elsevier. b) Schematic diagram of PMRs for the degradation/treatment of Toluene, acetone, chloroform, benzene, and xylene. Reproduced with permission.^[^ ^108^ ^]^ Copyright 2021, Elsevier. c) Schematic diagram of PMRs for the degradation/treatment of *n*‐hexane. Reproduced with permission.^[^ ^109^ ^]^ Copyright 2006, Elsevier. d) Schematic diagram of PMRs for the degradation/treatment of NO. Reproduced with permission.^[^ ^121^ ^]^ Copyright 2021, Elsevier. e) Schematic diagram of PMRs for the degradation/treatment of NO with a hybrid bioassisted process. Reproduced with permission.^[^ ^122^ ^]^ Copyright 2016, Elsevier. f) Schematic diagram of PMRs for the degradation/treatment of NO*_x_* with photocatalyst‐coated mixed matrix membrane arrangement. Reproduced with permission.^[^ ^123^ ^]^ Copyright 2022, American Chemical Society Publications.

Recently, G´erardin et al. performed *n*‐hexane removal in a lab‐scale PMR, as shown in **Figure** **6**a,^[^ ^107^ ^]^ whereby this hybrid plug‐flow system was designed to be integrated with a TiO_2_/polydimethylsiloxane (PDMS)/PAN membrane to evaluate the effect of different operating parameters, such as temperature, pressure, and composition, on the degradation and separation efficiency of *n*‐hexane. The system is equipped with three VOC feed ports for adjusting volumetric flow rates, a mixing chamber, a heater, a light source, a water source for humidification, and a condensate chamber for collecting condensates. Complete VOC removal was observed at low concentration (1–25 ppm) with low energy cost. The inclusion of a membrane module in the reactor significantly improved efficiency, even at low irradiance levels (≈3 W m^−2^), compared to a single plug‐flow reactor.

Similarly, Li et al. have incorporated a supported liquid membrane with photocatalysis in a batch reactor to degrade six different VOCs, as shown in Figure 6b.^[^ ^108^ ^]^ The equipment setup of this PMR is almost the same as that of the APMR01 discussed in Section 4, except for its sweep air and hot plate vaporization of VOCs. A removal efficiency of 60–80% was observed after 10 h of operation, and 25–55% of VOCs were mineralized at low concentrations of feed VOCs. This removal efficiency was enhanced by incorporating photocatalytic operation with a stable and highly active membrane module. Tsuru et al. have analyzed the degradation of methanol and ethanol on a modified TiO_2_‐supported ceramic membrane in a cylindrical PMR, as shown in Figure 6c.^[^ ^109^ ^]^ The reactor served as one of the earliest PMRs for removing VOCs from the air, with complete oxidation of methanol at concentrations up to 10 000 ppm. The arrangement resembled the standard PMR setup with conventional instruments, including a humidifier, a heater, and flow meters.

In another study, Su et al. developed a reactor with hierarchical nanostructured TiO_2_/PAN membranes for enhanced toluene elimination.^[^ ^110^ ^]^ Accordingly, the reactor with a TiO_2_/PAN mass ratio of 4/1 demonstrated the highest efficiency for toluene conversion at 97.4%. It has been established that the rate of toluene conversion increases when the TiO_2_/PAN mass ratio increases. Modesti et al. have developed PMR with various TiO_2_‐based photocatalytic membranes for methanol oxidation,^[^ ^81^ ^]^ where among the developed TiO_2_‐based membranes, the TiO_2_ nanofibers displayed better performance, with a conversion rate of ≈80%.

Hybrid photocatalytic membrane bioreactors (PMBRs) are gaining more attention in the discipline due to their hybrid photocatalytic for VOC treatment.^[^ ^111^, ^112^ ^]^ Various MBRs have been developed based on membrane bioreactors with *Pseudomonas putida*, including polyvinylidene fluoride hollow fiber with *Hydrogenophaga* for improved VOC treatment. For instance, Wei et al. have reported on N─TiO_2_/polypropylene (PP) PMBR for the toluene oxidation process,^[^ ^113^ ^]^ where this MBR successfully removed toluene with an efficiency of ≈99% and an elimination capacity of ≈550 g m^−3^ h^−1^ during an operational period of 500 days.

A continuous photocatalyst membrane biofilm reactor using a N─TiO_2_/PP membrane prepared via the sol–gel method demonstrated high efficiency in toluene degradation, achieving nearly 99% removal under visible light at a concentration of 140 mg m^−^^3^.^[^ ^114^ ^]^ Similarly, a batch Pyrex tubular reactor integrating graphene─TiO_2_/PVDF nanofiber membranes prepared through a solvothermal method and electrospinning successfully degraded methanol and acetaldehyde under UV light, achieving complete methanol degradation at a flow rate of 30 L min^−1^.^[^ ^115^ ^]^ The incorporation of graphene in the membrane structure facilitated improved electron transport, reducing charge recombination and enhancing photocatalytic efficiency.

Further, a cylindrical glass tube membrane module integrating porous TiO_2_/α‐alumina (α‐Al_2_O_3_) membranes prepared via the sol–gel process demonstrated enhanced methanol degradation at varying concentrations between 500 and 6000 ppmv under UV light.^[^ ^116^ ^]^ The inclusion of the membrane module increased the rate constant, highlighting the advantages of membrane‐assisted photocatalytic oxidation.

A modified cylindrical glass tube membrane module incorporating Pt‐modified porous TiO_2_/α‐Al_2_O_3_ membranes was also explored, effectively degrading methanol and ethanol at 100 ppm under UV light irradiation and achieving 100% removal efficiency.^[^ ^109^ ^]^ Meanwhile, a rectangular reactor integrating TiO_2_‐based membranes, including titanium silicate (TS‐1)/stainless steel (SS) and hybrid silicate (Sil‐1)/SS membranes, demonstrated a 52% removal efficiency for trichloroethylene at 240 ppmv.^[^ ^117^ ^]^ This study highlighted the role of hybrid silicate materials in enhancing VOC degradation.

A Pyrex tubular batch reactor equipped with five membrane modules using TiO_2_‐based membranes, including TiO_2_ nanofibers, nanocomposite membranes, and multilayer membranes, demonstrated varying methanol degradation efficiencies, with Type 1 achieving 80%, Type 2 achieving 40%, and Type 3 reaching 100% removal.^[^ ^81^ ^]^ Another study using a cylindrical laboratory‐scale water‐jacketed Pyrex glass reactor integrated TiO_2_/PP, WO_3_/PP, and TiO_2_/WO_3_/PP membranes for methane degradation at concentrations between 10 and 1000 ppm. The TiO_2_/WO_3_ blend exhibited the highest efficiency over a broad irradiance range.^[^ ^118^ ^]^ Additionally, a batch reactor using graphene oxide (GO)/manganese oxide (MnO*_x_*)/polymeric carbon nitride (CN) membranes efficiently removed formaldehyde, achieving over 90% degradation within 12 min of irradiation at an optimal catalyst loading ratio.^[^ ^119^ ^]^ A quartz cylindrical reactor integrating Pt/TiO_2_─ZnO membranes demonstrated a 74% removal efficiency for formaldehyde under UV–vis light in just 2 h.^[^ ^120^ ^]^ The research is summarized in Table 1 under VOC degradation applications.

#### 3.1.2 NO_x_ Treatment

Nitrogen oxides, such as nitrogen oxide and dioxide (NO and NO_2_), are considered significant pollutants as they are characteristic harmful inorganic compounds that are produced primarily due to fuel combustion from vehicles.^[^ ^124^, ^125^ ^]^ The transformation and utilization of light energy to activate the photocatalytic conversion of NO*_x_* under ambient settings has received considerable attention within the literature.^[^ ^126^, ^127^ ^]^

Recently, Liu et al. efficiently converted NO to nitrate ion (NO_3_^−^) in a reactor setup with PM (Si─TiO_2_ NR), as shown in Figure 6d.^[^ ^121^ ^]^ The arrangement was the same as APMR01, except for a steam inlet instead of air, a separate mixer, and constant‐temperature water circulation for absorption. The incorporation of a membrane module enhanced the overall efficiency (with removal of ≈67% and separation of ≈57%) of this PMR compared to individual PR systems.

Similarly, a schematic diagram of the PBMR using a Fe doping TiO_2_/polysulfone (FeTiO_2_/PSF) hybrid membrane for the efficient removal of NO is displayed in Figure 6e.^[^ ^122^ ^]^ The primary instrument and operation of this PBMR are similar to the APMR01 operation, with a different hybrid bioreactor design and bacterial nutrient tank and circulation. This integration of membrane photocatalysis with biodegradation achieved a removal efficiency of ≈86% for NO, with an elimination capacity of ≈160 gm^−3^ h^−1^. Additionally, compared to wet catalytic membrane reactors, the NO removal efficiency increased from 51.7% to 54.9%.

Photocatalytic cementitious materials in a membrane matrix are considered emergent materials for NO*_x_* removal under ambient circumstances.^[^ ^125^ ^]^ The research conducted by Pham et al. introduced a novel Ag/ZnO heterojunction catalyst coated on a polypropylene (PP) membrane, designed explicitly for the photocatalytic removal of NO*_x_* gas under visible light irradiation, as illustrated in Figure 6f.^[^ ^123^ ^]^ The incorporation of silver nanoparticles (3 wt%) enhanced the photocatalytic efficiency of ZnO through localized surface plasmon resonance, which promoted the separation of electron–hole pairs and reduced their recombination. The optimized Ag/ZnO/PP membrane demonstrated a NO removal efficiency of 46.52% while keeping the formation of NO_2_ at a low level. Stability tests indicated that the membrane maintained 40.87% efficiency after six cycles. Although the reactor system used in their study deviated slightly from the IUPAC definition of PMR, it was included in the discussion due to the similarity in the reaction and consecutive separation operations.

g‐C_3_N_4_ and 2D graphite‐like layered materials have garnered substantial attention as nonmetallic, visible‐light‐responsive photocatalysts for NO*_x_* degradation applications, outperforming traditional catalysts. Wang et al. have developed a PMR with g‐C_3_N_4_ microtubes prepared with an in situ soft‐chemical approach for the effective removal of NO.^[^ ^128^ ^]^ Furthermore, surface N‐vacancies were utilized as explicit sites for the adsorption and activation of NO, as well as for photoinduced electron capture, thereby enhancing the light‐absorbing capability of g‐C_3_N_4_. In addition, the porous wall‐structured g‐C_3_N_4_ microtubes facilitated reactant diffusion, with their tubular construction favoring the transfer of charge carriers. Lim et al. have demonstrated a hyper‐cross‐linked polymer with TiO_2_ nanoparticles toward NO*_x_* treatment.^[^ ^127^ ^]^ The capabilities and benefits of NO*_x_* treatment on epoxy─TiO_2_ aggregates were explored, focusing on the pores and coating of epoxy─TiO_2_ on both inner and outer surfaces.

In another study, Yang et al. prepared high‐chemical and thermal durability PCN‐based photocatalytic materials for the effective removal of NO*_x_*.^[^ ^129^ ^]^ It was observed that the ambient temperature, primary concentration of NO, and light intensity had a positive impact on the removal of NO*_x_*. Zhao et al. have further reported on concurrent SO_2_ and NO removal from flue gas at the TiO_2_‐based photocatalytic membrane under the UV irradiation strategy.^[^ ^125^ ^]^ Their results demonstrated that simultaneous desulfurization and denitrification were conducted on a TiO_2_‐based photocatalytic membrane, delivering a NO removal efficiency of ≈50%.

A single‐pass continuous flow reactor integrating nano‐TiO_2_/membrane filters (recycled fine glass without TiO_2_, RG‐T_0_ and RG with 2% TiO_2_, RG‐T_2_) through physical spreading achieved a NO*_x_* removal rate of 28 µmol m^2^ h⁻¹ under UV‐A light at 1000 ± 50 ppb.^[^ ^130^ ^]^ Another study employed a reactor designed according to International Organization for Standardization (ISO) Standard 22197‐1, integrating TiO_2_‐coated architectural membranes (photocatalytic materials (P1, P2) and control (C1)), which successfully removed NO*_x_* at 1000 ppb under UV‐A irradiation, with a peak removal rate of ≈1 µmol h⁻¹.^[^ ^131^ ^]^

A glass‐made cylindrical reactor incorporating hollow porous carbon nitride nanosphere coupled (HCNS)/rGO/carbonized polymer nanofiber (CNCF) membranes prepared via electrospinning achieved 64% NO removal under visible light at 600 ppb.^[^ ^132^ ^]^ Similarly, a batch photoreactor utilizing TiO_2_ nanoparticles embedded in PVDF/PDMS membranes demonstrated 100% NO removal under UV light.^[^ ^133^ ^]^ A self‐designed stainless‐steel batch photoreactor using TiO_2_ membranes prepared via hydrothermal treatment effectively removed NO at varying concentrations (628–1569 mg m^−^^3^) under UV light, with a removal rate of 67% at 25 °C. Additionally, a stainless‐steel reactor with a quartz window utilizing Si─TiO_2_/Al_2_O_3_ membranes prepared via hydrothermal treatment demonstrated 100% NO conversion in 11 min under UV light, highlighting the rapid reaction kinetics of this system.^[^ ^134^ ^]^

A rectangular stainless‐steel reactor integrated with Ag/ZnO/zinc/aluminum (ZnAL)‐layered double hydroxide (LDH) membranes demonstrated an enhanced NO removal efficiency of 46.53% at a Ag loading of 3% under visible light.^[^ ^135^ ^]^ Another study employed a PMR utilizing MOFs such as UiO‐66, UiO‐66─amino radical (NH_2_), Materials Institute Lavoisier (MIL)‐125, and MIL‐125─NH_2_ and demonstrated 100% NO*_x_* degradation under UV‐A light, irrespective of catalyst loading and initial NO*_x_* concentration.^[^ ^136^ ^]^ The research is summarized in Table 1 under NO*_x_* treatment applications.

## 4 Model PMR Design

**Figure 7:**
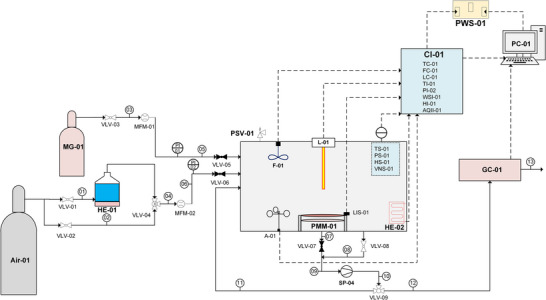
Process and instrumentation diagram (P&ID) of model PMR test system.

The theoretical model PMR (APMR01) design is focused on subjective investigation and can be used to develop practical test systems with basic equipment arrangements and layouts (**Figure** **7**). Additionally, this flow/equipment arrangement is flexible and can be used to test the effect of various design parameters (relative humidity (RH), temperature/pressure, airspeed, light intensity, and feed concentration) on the efficiency of PMR.

### 4.1 Operating Procedure

The process and instrumentation diagram (P&ID) for the model PMR is shown in Figure 7. Furthermore, **Table** **2** presents the instrument and its applications for the PMR model. As the table shows, model gas (MG) and air are separately input into the reactor zone, where the concentrations of MG and air are controlled using mass flow controllers (MFM‐01 and MFM‐02, respectively). The air is then subjected to a humidifier (THM‐01) for oxygen enrichment, and RH is controlled by the temperature of water/H_2_O_2_ and air inlet pressure. Dry air can be inputted to the reactor using bypass line 2. The reaction zone is presented as a cubic box for ease of presentation but can possess any shape (cylindrical, cubical, or rectangular) with desired dimensions, material (stainless steel, quartz, Pyrex, or glass), membrane unit (PMM‐01), and light source (L‐01) arrangement.

**Table 2:** Instrument description for model PMR.

Instrument name (number)	Application
Regulating instruments: fan (F‐01), heater (HE‐*n*), valves (VLV‐*n*), mass flow meters (MFM‐*n*), *n* = numbers labeled	Regulating reactor parameters such as indoor air circulation, temperature, and flow
Sensors (S): temperature (TS‐01), pressure (PS‐01), relative humidity (HS‐01), anemometer (A‐01)	Detection of values inside the reactor for each parameter
Indicators (I): TI‐01, PI‐*n*, wind speed (WSI‐01), HI‐01	Indication of parameter values
Controllers (C): TC‐01, fan speed (FC‐01), light switch (LC‐01)	Controlling components according to user set point
Pressure safety valve (PSV‐01)	Safety valve
Gas vacuum pump (SP‐01)	Regulating reactor pressure/ providing pressure gradient
Gas chromatography (GC‐01)	Detection of product components
Personal computer (PC‐01)	Master controller/recording, processing, and storing output values from different instruments
Power switch (PWS‐01)	Providing power to every instrument

### 4.2 Flow Passes and Configurations

Different flow arrangements can be used for both batch and continuous operations, offering flexibility through various flow passes. Accordingly, paths 7, 9, 10, and 11 are used to recycle gas to the reaction chamber for extra flow, where two to three flow configurations were mainly used in the research as best suited for testing purposes.^[^ ^109^, ^116^, ^117^ ^]^ In this design, two patterns are applied, with the first path following pathways 7, 9, 10, and 12, which can be used to analyze gases passed through PMM‐01 with or without photocatalysis (on/off L‐01). The second configuration follows along pathways 8, 9, 10, and 12, which can be implemented to analyze gases in the reaction chamber without the application of a membrane.

### 4.3 Safety

As harmful intermediates are formed during the reaction, the reaction chamber should be placed in the fume hood at all times in case of any leak or emergency opening of PSV‐01 due to pressure overload. Bypass lines equipped with fail‐open (white) or fail‐close (black) valves can be used to ensure the system's safety. The design presented here is primarily based on theoretical comparative analysis from various studies that inform and are presented in this review. Thus, for safety and other design considerations, it is recommended to follow guidelines established by the ISO standards for photocatalytic air purification (NO*_x_* and VOCs) with the addition of a membrane module.^[^ ^143^, ^144^ ^]^

## 5 Current Challenges and Future Trends in PMR Technology

Photocatalytic membrane technology represents novel solutions to address numerous current environmental issues.^[^ ^145^ ^]^ A synergistic strategy is implemented in PMR technology, where the incorporation of photocatalysis and molecular separation with membranes typically exceeds the procedural and practical limitations of distinct practices.^[^ ^144^ ^]^ However, PMRs have undergone some significant challenges that have hindered their laboratory and real‐world applications.

### 5.1 Challenges in Laboratory‐Scale PMRs

Although PMR design still provides reasonable fouling control, fouling is one of the most prominent problems that hinders PMR efficiency and thus deters PMR scale‐up.^[^ ^100^, ^146^ ^]^ For the effective operation of PMRs, it is essential to ensure that the degradation rate of air pollutants exceeds their adsorption rate, thus preventing membrane fouling issues. The balance between competitive adsorption and reactivity of gaseous reactants on the surface of photocatalytic membranes is another characteristic that must be optimized to mitigate fouling.^[^ ^147^ ^]^ Fouling is primarily related to the durability function of the membrane; therefore, membrane durability in the PMR unit is an additional reason for being conservative, one that limits its hands‐on usage. Moreover, forming a cake layer on the surface remains a challenge. Degraded adsorption of pollutants and photocatalysts at the surface of the membrane produces severe situations of entanglement and aperture hindrance, leading to reduced overall effectiveness of the inner layers.^[^ ^67^, ^100^ ^]^ This presents an opportunity to develop more novel approaches to address the challenges of membrane fouling. Additionally, large quantities of toxic solvents are used in the membrane engineering process, raising concerns about health and environmental impacts. Also, harmful activated intermediates may be produced if incomplete photocatalysis is performed in PMR.

PMR systems are facing a challenge from conventional reactor units, which may be more cost‐effective and time‐saving. Accordingly, UV light operations and reactant/product pumping are the primary sources of energy consumption, contributing to the overall operational cost. Hence, developing sunlight‐driven photocatalytic materials and durable membranes with advanced selectivity and permeability may moderate this problem. The primary point of discussion is the development of tailored photocatalytic membranes and membrane modules with low costs, durability in a wide range of operational settings, and high resistance to membrane fouling, thereby demonstrating outstanding recyclability and reproducibility.

Another challenge that researchers are currently facing involves attaching the photocatalyst to the membrane module in a coated membrane type to prevent photocatalytic leaching into the product stream. The preparation method consists of developing an intermediate layer with surface modifications to effectively attach the photocatalyst to the membrane surface.^[^ ^31^ ^]^ Moreover, PMR operation in the gas phase can be complex and is underrated when compared to the liquid phase in terms of degradation efficiency.^[^ ^148^ ^]^ Major environmental applications involve gas‐phase photocatalytic reactions and require reactive air species to degrade pollutants. There is a lower amount of hydroxyl reactive species in the air compared to water, which results in a decline in cost degradation efficiency.^[^ ^35^, ^149^ ^]^ Hence, humidification of the test feed is necessary to achieve better efficiency in air treatment. Additionally, an optimized PMR design for large‐scale environmental applications is highly desirable, where this can be achieved by optimizing design parameters such as the concentration and flow rate of reactants, photocatalyst loading, light source intensity, relative humidity, temperature, mode of operation (number of flow passes), and other factors.^[^ ^42^, ^150^ ^]^

Most current PMR studies have employed the utilization of simulated air/gas feed as a contamination source as a practical gas contaminants sample with ideal/controlled conditions. The primary factor that the source of practical gas samples comprises numerous other gases, particulate matter, and other air pollutants, which are relevant to real‐life situations, should not be overlooked, as they are likely to significantly influence the synergistic progression of the PMR.^[^ ^53^, ^151^ ^]^ Moreover, the availability of PMRs with substantial positive benefits on a broader scale is currently restricted to laboratory settings, which cannot be replicated in an engineering weighbridge.

#### 5.1.1 Photocatalytic Radical Effects on Polymer Membranes

One of the critical concerns in PMRs is the potential degradation of polymer membranes due to the generation of highly reactive radicals during photocatalytic reactions.^[^ ^89^ ^]^ Hydroxyl radicals (OH^•^) and superoxide anions (O_2_^−•^), which are essential for pollutant degradation, can also interact with polymeric membrane materials, leading to structural damage, loss of mechanical strength, and reduced membrane lifespan. This is particularly problematic for polymeric membranes such as PVDF, PES, and PAN, which may undergo oxidative degradation upon prolonged exposure to these radicals.^[^ ^35^ ^]^

Several strategies have been explored to prevent membrane degradation while maintaining high photocatalytic efficiency.^[^ ^38^ ^]^ One common approach is applying protective coatings on the membrane surface, such as thin oxide layers (SiO_2_, Al_2_O_3_, ZrO_2_) or carbon‐based coatings (graphene oxide, reduced graphene oxide), which act as a physical barrier to prevent direct contact between the polymer membrane and reactive species. Another strategy is to modify photocatalysts to control radical generation. Heterojunction photocatalysts and noble metal doping can help regulate the reactivity of radicals, directing their action toward pollutants rather than the membrane surface. Additionally, radical scavengers such as ascorbic acid or organic functional groups can be introduced into the membrane matrix to neutralize excess radicals before they cause damage.

Recent studies have demonstrated that composite membranes incorporating ceramic or carbon‐based reinforcements exhibit enhanced durability under photocatalytic conditions.^[^ ^152^ ^]^ For instance, the use of TiO_2_─PVDF hybrid membranes with an intermediate protective SiO_2_ layer has shown significant resistance to radical‐induced degradation.^[^ ^153^ ^]^ Similarly, GO‐modified PES membranes have exhibited higher oxidative resistance due to the protective function of graphene oxide, which prevents direct exposure of the polymer surface to radicals.^[^ ^154^ ^]^

Future research should focus on developing self‐healing membranes that can repair minor oxidative damage autonomously and optimize radical management strategies to ensure the long‐term viability of PMRs in real‐world applications. By addressing these concerns, the stability and durability of polymeric membranes in PMRs can be significantly improved, making them more suitable for large‐scale air purification applications.

#### 5.1.2 Challenges in Industrial‐Scale PMRs

Despite the growing research in lab‐scale photocatalytic technologies for air purification, large‐scale studies on PMRs remain scarce. While numerous studies have explored photocatalytic reactors for air treatment, large‐scale applications of PMRs in this field are limited.^[^ ^50^, ^155^ ^]^ By contrast, large‐scale PMRs have been more commonly used in water and wastewater treatment, where the operating conditions are more favorable for efficient photocatalysis.^[^ ^156^ ^]^

One of the key technical challenges in air‐based PMRs is the limited generation of OH^•^ radicals, which is crucial for photocatalytic oxidation.^[^ ^4^, ^157^ ^]^ In water treatment, an aqueous medium provides a continuous source of water molecules, which act as electron donors and sustain a high concentration of hydroxyl radicals. By contrast, in air‐phase systems, the absence of a liquid phase significantly reduces OH^•^ radical production, thereby limiting degradation efficiency. While high humidity can enhance OH^•^ formation, maintaining an optimal humidity level in large‐scale industrial environments is challenging, as airflows, temperature variations, and pressure changes make it difficult to sustain consistent moisture levels. While laboratory settings allow for controlled humidity, real‐world industrial applications introduce fluctuations that reduce efficiency.^[^ ^158^ ^]^

Another vital concern is inadequate UV activation in large‐scale setups. Photocatalysis relies on light activation, typically in the UV‐A range (≈365 nm) or visible spectrum for advanced catalysts. However, in large‐scale PMRs, ensuring uniform and efficient UV light penetration across the membrane surface becomes problematic. Industrial reactors require high‐powered UV lamps; however, light intensity decreases significantly with distance due to the inverse square law, resulting in uneven catalyst activation in large systems. Additionally, UV sources in industrial settings consume substantial energy, making operational costs a significant concern.^[^ ^159^ ^]^

Beyond efficiency concerns, UV system safety is another significant barrier to large‐scale PMR adoption. UV safety measures can be strictly controlled in laboratory environments through the use of shielding, enclosures, and automated shutoff systems.^[^ ^160^ ^]^ However, in industrial‐scale settings, exposure to high‐intensity UV radiation poses potential health risks to workers, including eye damage and skin burns. Large‐scale PMR systems would require specialized UV shielding, safety training, and operational protocols, further increasing costs and complexity. The potential safety hazards make industries hesitant to adopt PMR‐based air treatment over safer, more conventional alternatives such as electrostatic precipitators or activated carbon filtration.^[^ ^161^ ^]^

Furthermore, mass transfer limitations present another barrier to large‐scale implementation. In water treatment, pollutants are in direct contact with the photocatalyst due to high solubility and diffusion rates, facilitating effective degradation. However, in air‐phase reactions, gas‐phase pollutants have lower diffusivity and solubility, leading to reduced interaction with the photocatalytic membrane surface. Ensuring sufficient residence time and contact between air pollutants and the catalyst becomes increasingly difficult at larger scales, requiring complex reactor designs to enhance turbulence and adsorption—further increasing costs and engineering challenges.^[^ ^162^ ^]^

Membrane fouling and long‐term stability are also significant concerns. Backwashing, chemical cleaning, and cross‐flow filtration in water treatment help mitigate fouling, extending the membrane's lifespan. In air purification, however, dry‐phase fouling, caused by dust, soot, and organic films, as well as catalyst deactivation, leads to performance degradation over time.^[^ ^163^ ^]^ Industrial air environments are often harsh and characterized by high concentrations of particulate matter, corrosive gases, and fluctuating conditions, all of which contribute to accelerated membrane degradation. Traditional photocatalysts, such as TiO₂, can suffer from deactivation due to surface contamination, necessitating frequent regeneration or replacement, which increases maintenance costs and limits long‐term feasibility.^[^ ^54^ ^]^

Finally, the cost‐effectiveness of PMRs in large‐scale air treatment remains a significant challenge. Compared to conventional air purification methods such as activated carbon filtration, electrostatic precipitators, and biofilters, PMRs require substantial initial investment, high operational energy costs (due to UV activation), and ongoing maintenance.^[^ ^164^ ^]^ By contrast, PMRs in water treatment are more cost‐efficient because the liquid phase enhances photocatalytic activity, reaction kinetics are faster, and pollutant concentrations are higher, making the technology more economically viable.^[^ ^165^ ^]^

As a result, while PMRs have shown great potential in water and wastewater treatment, their widespread adoption in large‐scale air purification remains limited due to technical barriers related to hydroxyl radical generation, UV activation challenges, safety concerns, mass transfer inefficiencies, membrane fouling, long‐term stability issues, and high operational costs. Addressing these challenges will require significant advancements in catalyst design, reactor engineering, energy‐efficient UV systems, and enhanced safety protocols before PMRs can become viable for industrial‐scale air treatment applications.^[^ ^165^, ^166^ ^]^

**Figure 8:**
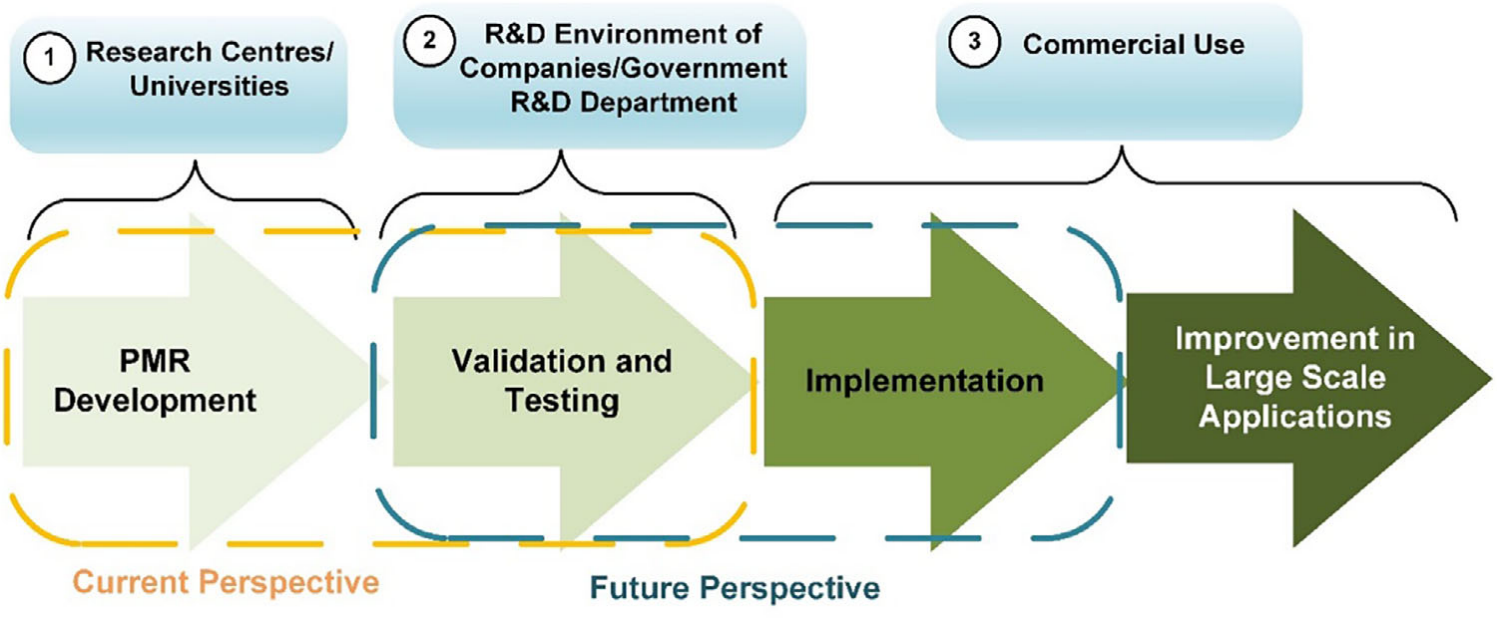
Progress in PMR technology development.

The current progress and future prospects of PMR technology are presented in an overview in **Figure** **8**. Due to the increasingly severe energy crisis and environmental pollution challenges, novel designs for enhancing the sustainability of photocatalytic membrane technology and promoting green transformation in this field of engineering are imperative.^[^ ^167^ ^]^ Significant developments have occurred in the sculpting and imitation of PMR, expanding activity and durability through the appropriate design of the reactor, operational settings, and systematic study of their structural and functional relationships.^[^ ^168^ ^]^ Thus, additional research effort in this field is essential, set at a pilot level, with practical air trials for pavement, a tactic to scale‐up PMR systems. For example, the feasible adoption of membrane‐derived photocatalysis, integrated with other novel techniques such as biodegradation and plasma, offers a new strategy for air pollution control.^[^ ^26^, ^169^ ^]^ Likewise, computer‐aided strategies such as computational fluid dynamics can be applied to the study and development of PMR designs to simulate fluid flows and analyze reactant characteristics within a system, enhancing the improvement process. Additionally, advanced strategies are required to develop visible light photoactive materials with modified properties and effective nanoscale irregularity, which are crucial in designing flexible and robust photocatalysts/membranes for use in PMR systems. Therefore, it is essential to develop key components to enhance novel membranes and nanostructured photocatalysts that can overcome the aforementioned challenges. These challenges include the need for high resilience and long‐term stability to withstand physical and chemical stresses.

Furthermore, to make PMR technology practically viable, intricate work is required to prepare novel hybrid PMs, model and simulate reactant and product distributions, and design PMR reaction zones that incorporate real‐life conditions. This domain has considerable untapped potential toward developing a dependable and economically feasible system for controlling air pollution.

Moreover, future research should focus on developing next‐generation photocatalysts with improved stability, reusability, and visible‐light activity. Innovations in PM materials with enhanced photocatalyst integration, durability, and self‐cleaning properties will further improve efficiency. Optimizing PMR reactor designs to maximize pollutant contact, minimize fouling, and improve energy efficiency will be crucial in making these systems more viable for real‐world applications. Additionally, exploring scalable fabrication techniques for cost‐effective large‐scale production of PMRs will be essential in transitioning these technologies from laboratory studies to industrial applications. By addressing these challenges, PMRs can become a commercially viable solution for sustainable air and water purification.

## 6 Conclusion

Recent advancements and critical analysis of PMR in gas‐phase air pollution control, including VOC degradation and NO*_x_* treatment, are highlighted in this review. PMR is a promising technology for air treatment, in which membrane‐based photocatalytic architectures enable the synergistic effect of both reaction and separation in a single step, resulting in enhanced degradation efficiency due to improved charge‐transport properties in the photocatalytic reaction step. Additionally, the spatial incorporation of membrane‐bound components/materials reduces the effect of charge recombination, which is crucial in determining the quantum efficiency of ionic and electronic transport processes in photocatalytic membrane systems. The integration strategies established in PMR development more broadly reveal new approaches for controlling membrane fouling, the activity of photocatalysts, gas selectivity, and the rejection rate of the membrane. The model design of PMR is presented based on numerous theoretical studies with detailed instrumentation, operation, and safety features. Current challenges in PMR systems, such as photocatalyst deactivation, membrane fouling, and a decline in photocatalytic efficiency, are also analyzed. Furthermore, brief descriptions are provided of outlooks on upcoming research and development opportunities for state‐of‐the‐art PMR technology.

## Conflict of Interest

The authors declare no conflict of interest.

## References

[1] M. N. Azimi , M. M. Rahman , Sci. Rep. 2024, 14, 9856. 38684837 10.1038/s41598-024-60786-0 PMC11058277

[2] R. Chandrappa , U. C. Kulshrestha , in Sustainable Air Pollution Management, Springer, Berlin, Heidelberg 2016, pp. 325–343.

[3] X. Liu , R. J. Koestler , T. Warscheid , Y. Katayama , J.‐D. Gu , Nat. Sustainability 2020, 3, 991.

[4] M. Brusseau , A. Matthias , A. Comrie , S. Musil , in Environmental and Pollution Science, Elsevier, Amsterdam, Netherlands 2019, pp. 293–309.

[5] H. Chojer , P. T. B. S. Branco , F. G. Martins , M. C. M. Alvim‐Ferraz , S. I. V. Sousa , Environ. Technol. Innovation 2024, 33, 103534.

[6] X.‐C. Wang , J. J. Klemeš , X. Dong , W. Fan , Z. Xu , Y. Wang , P. S. Varbanov , Renewable Sustainable Energy Rev. 2019, 105, 71.

[7] A. Bhatnagar , Annu. Rev. Med. 2022, 73, 393. 34644154 10.1146/annurev-med-042220-011549 PMC10132287

[8] World Health Organization, *Air Pollution* , 2024, https://www.who.int/health‐topics/air‐pollution#tab=tab_1 (accessed: April 2025).

[9] F. Capozzi , A. Di Palma , P. Adamo , M. Sorrentino , S. Giordano , V. Spagnuolo , Environ. Pollut. 2019, 249, 566. 30933753 10.1016/j.envpol.2019.03.012

[10] A. M. Lai , E. Carter , M. Shan , K. Ni , S. Clark , M. Ezzati , C. Wiedinmyer , X. Yang , J. Baumgartner , J. J. Schauer , Sci. Total Environ. 2019, 646, 309. 30055493 10.1016/j.scitotenv.2018.07.322

[11] M. Secrest , J. Schauer , E. Carter , J. Baumgartner , Indoor Air 2017, 27, 1052. 28401994 10.1111/ina.12389

[12] K. K. Meena , A. K. Goswami , Transp. Policy 2024, 154, 48.

[13] S. Vardoulakis , E. Giagloglou , S. Steinle , A. Davis , A. Sleeuwenhoek , K. S. Galea , K. Dixon , J. O. Crawford , Int. J. Environ. Res. Public Health 2020, 17, 8972. 33276576 10.3390/ijerph17238972 PMC7729884

[14] B. E. Cummings , M. S. Waring , J. Exposure Sci. Environ. Epidemiol. 2020, 30, 253. 10.1038/s41370-019-0175-9 31695112

[15] Z. Klimont , C. Heyes , P. Rafaj , L. Höglund‐Isaksson , P. Purohit , K. Kaltenegger , A. Gomez‐Sanabria , Y. Kim , W. Winiwarter , L. Warnecke , W. Schoepp , F. Lindl , G. Kiesewetter , R. Sander , B. Nguyen , Global Gridded Anthropogenic Emissions of Air Pollutants and Methane for the Period 1990–2050, Zenodo 2023, 10.5281/zenodo.10366132.

[16] NASA Goddard Institute for Space Studies, *Panoply Data Viewer* , 2024, https://www.giss.nasa.gov/tools/panoply/ (accessed: April 2025).

[17] K. M. Emmerson , M. D. Keywood , in Australia State of the Environment 2021, Australian Government Department of Agriculture, Water and the Environment, Canberra 2021.

[18] L. Zhou , C. Ma , J. Horlyck , R. Liu , J. Yun , Catalysts 2020, 10, 668.

[19] M. Meena , P. Sonigra , G. Yadav , Environ. Sci. Pollut. Res. 2021, 28, 2485. 10.1007/s11356-020-11112-4 33095900

[20] Y. Guo , M. Wen , G. Li , T. An , Appl. Catal., B 2021, 281, 119447.

[21] A. Luengas , A. Barona , C. Hort , G. Gallastegui , V. Platel , A. Elias , Rev. Environ. Sci. Bio/Technol. 2015, 14, 499.

[22] R. K. Patel , A. P. Singh , R. Shankar , P. Khare , A. K. Thakur , V. L. Gole , Int. J. Chem. React. Eng. 2023, 22, 11.

[23] T. Chen , C. Fu , Y. Liu , F. Pan , F. Wu , Z. You , J. Li , Sep. Purif. Technol. 2021, 264, 118464.

[24] A. O. Ibrahim , K. A. Adegoke , R. O. Adegoke , Y. A. AbdulWahab , V. B. Oyelami , M. O. Adesina , J. Mol. Liq. 2021, 333, 115593.

[25] M. Galle , D. Agar , O. Watzenberger , Chem. Eng. Sci. 2001, 56, 1587.

[26] V. K. H. Bui , T. N. Nguyen , V. Van Tran , J. Hur , I. T. Kim , D. Park , Y.‐C. Lee , Environ. Technol. Innovation 2021, 22, 101471.

[27] Y. Huang , S. S. H. Ho , Y. Lu , R. Niu , L. Xu , J. Cao , S. Lee , Molecules 2016, 21, 56. 26742024 10.3390/molecules21010056 PMC6273848

[28] H. Huang , G. Liu , Y. Zhan , Y. Xu , H. Lu , H. Huang , Q. Feng , M. Wu , Catal. Today 2017, 281, 649.

[29] V. Soni , V. Goel , P. Singh , A. Garg , Int. J. Chem. React. Eng. 2021, 19, 1.

[30] Photocatalysis: Fundamental Processes and Applications, Vol. 32 (Ed: S. L. Suib ), Elsevier, Amsterdam 2021, pp. 761–790.

[31] R. Ahmad , Z. Ahmad , A. U. Khan , N. R. Mastoi , M. Aslam , J. Kim , J. Environ. Chem. Eng. 2016, 4, 4143.

[32] Y. Zhao , Y. Wei , X. Wu , H. Zheng , Z. Zhao , J. Liu , J. Li , Appl. Catal., B 2018, 226, 360.

[33] D. L. Rocha , M. Y. Kamogawa , F. R. Rocha , Anal. Chim. Acta 2015, 896, 11. 26481985 10.1016/j.aca.2015.09.027

[34] W. A. Jacoby , D. M. Blake , J. A. Penned , J. E. Boulter , L. M. Vargo , M. C. George , S. K. Dolberg , J. Air Waste Manage. Assoc. 1996, 46, 891. 10.1080/10473289.1996.10467525 28081402

[35] P. Argurio , E. Fontananova , R. Molinari , E. Drioli , Processes 2018, 6, 162.

[36] X. Yan , J. Li , C. Ma , Y. Tang , X. Kong , J. Lu , Water Sci. Technol. 2020, 81, 131. 32293596 10.2166/wst.2020.091

[37] C. N. Rani , S. Karthikeyan , Water Sci. Technol. 2018, 77, 2642. 29944129 10.2166/wst.2018.220

[38] M. Romay , N. Diban , M. J. Rivero , A. Urtiaga , I. Ortiz , Catalysts 2020, 10, 570.

[39] F. Gallucci , A. Basile , F. I. Hai , in Membranes for Membrane Reactors: Preparation, Optimization and Selection (Eds.: A. Basile , F. Gallucci ), Wiley, Chichester 2011, pp. 1–61.

[40] V. Palma , D. Barba , E. Meloni , C. Ruocco , M. Martino , in Current Trends and Future Developments on (Bio‐) Membranes, Elsevier, Amsterdam, Netherlands 2020, pp. 33–56.

[41] M. Li , B. Lu , Q.‐F. Ke , Y.‐J. Guo , Y.‐P. Guo , J. Hazard. Mater. 2017, 333, 88. 28342359 10.1016/j.jhazmat.2017.03.019

[42] X. Zheng , Z.‐P. Shen , L. Shi , R. Cheng , D.‐H. Yuan , Catalysts 2017, 7, 224.

[43] S. Mozia , Sep. Purif. Technol. 2010, 73, 71.

[44] J. Zhang , S. Zhang , C. Peng , Y. Chen , Z. Tang , Q. Wu , React. Chem. Eng. 2020, 5, 2250.

[45] M. Rajca , Chem. Eng. J. 2016, 305, 169.

[46] R. Goei , T.‐T. Lim , Ceram. Int. 2014, 40, 6747.

[47] D. S. Selishchev , T. N. Filippov , M. N. Lyulyukin , D. V. Kozlov , Chem. Eng. J. 2019, 370, 1440.

[48] D. Robert , V. Keller , N. Keller , in Photocatalysis and Water Purification: From Fundamentals to Recent Applications, (Ed.: P. Pichat ), Wiley‐VCH, Weinheim 2013, pp. 145–178.

[49] V. Augugliaro , G. Palmisano , L. Palmisano , J. Soria , in Heterogeneous Photocatalysis: Relationships with Heterogeneous Catalysis and Perspectives, (Eds.: G. Marcì , L. Palmisano ), Elsevier, Amsterdam 2019, pp. 1–24.

[50] C. Chen , L. Fei , B. Wang , J. Xu , B. Li , L. Shen , H. Lin , Small 2024, 20, 2305066.

[51] N. S. Mabaso , C. S. Tshangana , A. A. Muleja , Membranes 2024, 14, 217. 39452829 10.3390/membranes14100217 PMC11509138

[52] L. Luo , Q. Zhao , Y. Yang , T. Wu , M. Qiang , W. Que , Adv. Sustainable Syst. 2025, 9, 2401024.

[53] T.‐T. Lim , P.‐S. Yap , M. Srinivasan , A. G. Fane , Crit. Rev. Environ. Sci. Technol. 2011, 41, 1173.

[54] S. Riaz , S.‐J. Park , J. Ind. Eng. Chem. 2020, 84, 23.

[55] S. Mozia , M. Tomaszewska , A. W. Morawski , Appl. Catal., B 2005, 59, 131.

[56] J. Wu , Y. Alipouri , H. Luo , L. Zhong , J. Hazard. Mater. 2022, 421, 126766. 34396962 10.1016/j.jhazmat.2021.126766

[57] R. Molinari , L. Palmisano , V. Loddo , S. Mozia , A. Morawski , in Handbook of Membrane Reactors, Elsevier, Amsterdam, Netherlands 2013, pp. 808–845.

[58] M. R. Rahimpour , L. Mahmoodi , in Current Trends and Future Developments on (Bio‐) Membranes, Elsevier, Amsterdam, Netherlands 2018, pp. 173–188.

[59] C. Karthikeyan , P. Arunachalam , K. Ramachandran , A. M. Al‐Mayouf , S. Karuppuchamy , J. Alloys Compd. 2020, 828, 154281.

[60] G. Camera‐Roda , V. Loddo , L. Palmisano , F. Parrino , F. Santarelli , Chem. Eng. J. 2017, 310, 352.

[61] R. Molinari , P. Argurio , K. Szymański , D. Darowna , S. Mozia , in Current Trends and Future Developments on (Bio‐) Membranes: Membrane Technology for Water and Wastewater Treatment – Advances and Emerging Processes (Ed.: A. Basile ), Elsevier, Amsterdam 2020, pp. 83–116.

[62] D. Zhang , G. Li , C. Y. Jimmy , J. Mater. Chem. 2010, 20, 4529.

[63] A. Bhattacharya , S. Ambika , in >Membrane Based Methods for Dye Containing Wastewater (Eds.: S. S. Muthu , A. Khadir ), Springer, Singapore 2022, pp. 49–77.

[64] J. Hu , Y. Ji , Z. Mo , N. Li , Q. Xu , Y. Li , H. Xu , D. Chen , J. Lu , J. Mater. Chem. A 2019, 7, 4408.

[65] R. Molinari , C. Lavorato , P. Argurio , Catal. Today 2017, 281, 144.

[66] R. Molinari , C. Lavorato , P. Argurio , Catalysts 2021, 11, 775.

[67] H. Zhang , Y. Wan , J. Luo , S. B. Darling , ACS Appl. Mater. Interfaces 2021, 13, 14844. 33769034 10.1021/acsami.1c01131

[68] A. Murali , H. Sohn , J. Nanosci. Nanotechnol. 2019, 19, 4377. 30913728 10.1166/jnn.2019.16363

[69] M. Mitra , A. Ghosh , A. Mondal , K. Kargupta , S. Ganguly , D. Banerjee , Appl. Surf. Sci. 2017, 402, 418.

[70] Y. Li , C. Gao , R. Long , Y. Xiong , Mater. Today Chem. 2019, 11, 197.

[71] L. Zhong , F. Haghighat , Build. Environ. 2015, 91, 191.

[72] M. Al‐Akhali , M. S. Chaar , A. Elsayed , A. Samran , M. Kern , J. Mech. Behav. Biomed. Mater. 2017, 74, 245. 28633093 10.1016/j.jmbbm.2017.06.013

[73] J. Bill , D. Heimann , J. Eur. Ceram. Soc. 1996, 16, 1115.

[74] M. Chai , W. Tong , Z. Wang , S. Zhao , Y. Zhang , Macromol. Mater. Eng. 2022, 307, 2100753.

[75] X. Cheng , H. Liang , F. Qu , A. Ding , H. Chang , B. Liu , X. Tang , D. Wu , G. Li , Chem. Eng. J. 2017, 308, 1010.

[76] W. J. Lee , Y. Bao , C. Guan , X. Hu , T.‐T. Lim , Chem. Eng. J. 2021, 410, 128307.

[77] U. Bellè , M. Invernizzi , E. Polvara , A. Lucotti , M. V. Diamanti , S. Sironi , M. Pedeferri , Chem. Eng. J. 2022, 437, 135323.

[78] T. M. Fujimoto , M. Ponczek , U. L. Rochetto , R. Landers , E. Tomaz , Environ. Sci. Pollut. Res. 2017, 24, 6390. 10.1007/s11356-016-6494-7 27026546

[79] Y.‐H. Li , S.‐W. Cheng , C.‐S. Yuan , T.‐F. Lai , C.‐H. Hung , Chemosphere 2018, 208, 808. 29906755 10.1016/j.chemosphere.2018.06.035

[80] M. V. Diamanti , M. Ormellese , M. Pedeferri , J. Exp. Nanosci. 2015, 10, 1285.

[81] M. Modesti , M. Roso , C. Boaretti , S. Besco , D. Hrelja , P. Sgarbossa , A. Lorenzetti , Appl. Catal., B 2014, 144, 216.

[82] S. Bai , X. Wang , C. Hu , M. Xie , J. Jiang , Y. Xiong , Chem. Commun. 2014, 50, 6094. 10.1039/c4cc00745j 24777281

[83] Z. Tong , D. Yang , J. Shi , Y. Nan , Y. Sun , Z. Jiang , ACS Appl. Mater. Interfaces 2015, 7, 25693. 26545166 10.1021/acsami.5b09503

[84] X. Zhang , X. Xie , H. Wang , J. Zhang , B. Pan , Y. Xie , J. Am. Chem. Soc. 2013, 135, 18. 23244197 10.1021/ja308249k

[85] H. Zheng , Z. Zhao , J. B. Phan , H. Ning , Q. Huang , R. Wang , J. Zhang , W. Chen , ACS Appl. Mater. Interfaces 2019, 12, 2145. 31845568 10.1021/acsami.9b19915

[86] N. He , S. Cao , J. Gu , A. Uddin , C. Zhang , Y. Yu , H. Chen , F. Jiang , J. Colloid Interface Sci. 2022, 606, 1284. 34492466 10.1016/j.jcis.2021.08.122

[87] H. Gao , R. Cao , S. Zhang , H. Yang , X. Xu , ACS Appl. Mater. Interfaces 2018, 11, 2050. 10.1021/acsami.8b17757 30561185

[88] Z. L. Xu , F. A. Qusay , J. Appl. Polym. Sci. 2004, 91, 3398.

[89] S. Kundu , N. Karak , Chem. Eng. J. 2022, 438, 135575.

[90] Y. Qing , Y. Li , Z. Guo , Y. Yang , W. Li , J. Environ. Chem. Eng. 2022, 10, 108648.

[91] F. Bi , Z. Zheng , R. Li , R. Du , L. Zhao , S. Xiao , L. Wang , X. Dong , Chem. Eng. J. 2025, 507, 160781.

[92] J. Liu , Q. Liao , C. Li , X. Jiang , J. Li , W. Jin , G. Liu , Y. Guo , B. Yu , H. Zhu , Process Saf. Environ. Prot. 2025, 193, 940.

[93] S. A. Zikalala , O. T. Mahlangu , J. Li , B. B. Mamba , E. N. Nxumalo , J. Appl. Polym. Sci. 2025, 142, 56704.

[94] N. Morante , G. Viscusi , G. Gorrasi , K. Monzillo , D. Sannino , J. Water Process Eng. 2025, 69, 106529.

[95] F. E. Bortot Coelho , S. I. Sohn , V. M. Candelario , N. I. B. Hartmann , C. Hélix‐Nielsen , W. Zhang , J. Membr. Sci. 2025, 715, 123485.

[96] S. Li , B.‐K. Lee , Sep. Purif. Technol. 2025, 364, 132479.

[97] S. Mohd Saleh , P. C. Oh , M. M. Zain , V. C. Quek , A. S. Zulkifli , T. L. Chew , J. Ind. Eng. Chem. 2024, 131, 230.

[98] B. I. Waisi , S. S. Manickam , N. E. Benes , A. Nijmeijer , J. R. McCutcheon , Ind. Eng. Chem. Res. 2019, 58, 4084.

[99] R. Molinari , T. Marino , P. Argurio , Int. J. Hydrogen Energy 2014, 39, 7247.

[100] W. Zhang , L. Ding , J. Luo , M. Y. Jaffrin , B. Tang , Chem. Eng. J. 2016, 302, 446.

[101] M. Pidou , S. A. Parsons , G. Raymond , P. Jeffrey , T. Stephenson , B. Jefferson , Water Res. 2009, 43, 3932. 19539972 10.1016/j.watres.2009.05.030

[102] M. Birnie , S. Riffat , M. Gillott , Int. J. Low‐Carbon Technol. 2006, 1, 47.

[103] W. R. Chen , C. M. Sharpless , K. G. Linden , I. Suffet , Environ. Sci. Technol. 2006, 40, 2734. 16683616 10.1021/es051961m

[104] R. Montero‐Montoya , R. López‐Vargas , O. Arellano‐Aguilar , Ann. Glob. Health 2018, 84, 225. 30873816 10.29024/aogh.910 PMC6748254

[105] D. R. Widiana , Y.‐F. Wang , S.‐J. You , H.‐H. Yang , L.‐C. Wang , J.‐H. Tsai , H.‐M. Chen , Aerosol Air qual. Res. 2019, 19, 375.

[106] A. C. Heeley‐Hill , S. K. Grange , M. W. Ward , A. C. Lewis , N. Owen , C. Jordan , G. Hodgson , G. Adamson , Environ. Sci.: Processes Impacts 2021, 23, 699. 10.1039/d0em00504e 34037627

[107] F. Gérardin , A. Cloteaux , J. Simard , É. Favre , Chem. Eng. J. 2021, 419, 129566.

[108] J. Li , B. Li , G. Sui , L. Du , Y. Zhuang , Y. Zhang , Y. Zou , J. Mol. Struct. 2021, 1231, 130023.

[109] T. Tsuru , T. Kan‐no , T. Yoshioka , M. Asaeda , J. Membr. Sci. 2006, 280, 156.

[110] J. Su , G. Yang , C. Cheng , C. Huang , H. Xu , Q. Ke , J. Colloid Interface Sci. 2017, 507, 386. 28806658 10.1016/j.jcis.2017.07.104

[111] S. Leong , A. Razmjou , K. Wang , K. Hapgood , X. Zhang , H. Wang , J. Membr. Sci. 2014, 472, 167.

[112] L. Qin , Y. Zhang , Z. Xu , G. Zhang , Bioresour. Technol. 2018, 269, 476. 30139558 10.1016/j.biortech.2018.08.062

[113] Z. Wei , Y. He , Z. Huang , X. Xiao , B. Li , S. Ming , X. Cheng , Ecotoxicol. Environ. Saf. 2019, 184, 109618. 31487569 10.1016/j.ecoenv.2019.109618

[114] Z. S. Wei , Y. M. He , Z. S. Huang , X. L. Xiao , B. L. Li , S. Ming , X. L. Cheng , Ecotoxicol. Environ. Saf. 2019, 184, 109618. 31487569 10.1016/j.ecoenv.2019.109618

[115] C. Boaretti , G. Vitiello , G. Luciani , A. Lorenzetti , M. Modesti , M. Roso , Catalysts 2020, 10, 1017.

[116] T. Tsuru , T. Kan‐no , T. Yoshioka , M. Asaeda , Catal. Today 2003, 82, 41.

[117] A. J. Maira , W. N. Lau , C. Y. Lee , P. L. Yue , C. K. Chan , K. L. Yeung , Chem. Eng. Sci. 2003, 58, 959.

[118] I. R. Bellobono , F. Groppi , M. Sturini , A. Albini , F. Morazzoni , J. Chem. Chem. Eng. 2020, 14, 73.

[119] Z. Wang , H. Yu , Y. Xiao , L. Zhang , L. Guo , L. Zhang , X. Dong , Chem. Eng. J. 2020, 394, 125014.

[120] C. Nie , L. Liu , R. He , Sep. Purif. Technol. 2018, 206, 316.

[121] W. Liu , Y. Yun , M. Li , J. Mao , C. Li , C. Li , L. Hu , Chem. Eng. J. 2022, 437, 135261.

[122] Z. S. Wei , B. R. Li , J. B. Wang , Z. S. Huang , Y. M. He , Z. Y. Chen , Chem. Eng. J. 2016, 296, 154.

[123] V. V. Pham , H. P. Phan La , H. V. Le , S. T. Truong , T. Q. Nguyen , T. M. Cao , Ind. Eng. Chem. Res. 2022, 61, 12427.

[124] V. Muñoz , C. Casado , S. Suárez , B. Sánchez , J. Marugán , Catal. Today 2019, 326, 82.

[125] Y. Zhao , J. Han , Y. Shao , Y. Feng , Environ. Technol. 2009, 30, 1555. 20184000 10.1080/09593330903313786

[126] R. Zouzelka , J. Rathousky , Appl. Catal., B 2017, 217, 466.

[127] T. Lim , J. H. Lee , J.‐H. Mun , K.‐H. Yang , S. Ju , S.‐M. Jeong , Polymers 2020, 12, 2384. 33081225 10.3390/polym12102384 PMC7602755

[128] Z. Wang , Y. Huang , M. Chen , X. Shi , Y. Zhang , J. Cao , W. Ho , S. C. Lee , ACS Appl. Mater. Interfaces 2019, 11, 10651. 30807084 10.1021/acsami.8b21987

[129] Y. Yang , T. Ji , Y. Lin , W. Su , J. Cleaner Prod. 2021, 295, 126458.

[130] M.‐Z. Guo , J. Chen , M. Xia , T. Wang , C. S. Poon , Build. Environ. 2018, 144, 412.

[131] X. Tang , O. Rosseler , S. Chen , S. Houzé de l'Aulnoit , M. J. Lussier , J. Zhang , G. Ban‐Weiss , H. Gilbert , R. Levinson , H. Destaillats , Appl. Catal., B 2021, 281, 119260.

[132] H. Wu , D. Chen , N. Li , Q. Xu , H. Li , J. He , J. Lu , Nanoscale 2016, 8, 12066. 27245319 10.1039/c6nr02955h

[133] R. Majidi , J. Parhizkar , E. Karamian , Nanochem. Res. 2018, 3, 212.

[134] W. Liu , C. Li , J. Mao , L. Hu , M. Li , Y. Yun , C. Lu , Sep. Purif. Technol. 2023, 318, 123928.

[135] H. Tang , X. Luo , W. Li , Y. Pan , S. Wang , H. Ma , Y. Shen , R. Fang , F. Dong , Chem. Eng. J. 2023, 474, 145873.

[136] J. Teixeira , P. M. Martins , R. F. de Luis , E. Falletta , M. F. Ordoñez , C. L. Bianchi , S. Lanceros‐Méndez , Chem. Eng. J. 2024, 486, 150400.

[137] M. Roso , A. Lorenzetti , C. Boaretti , D. Hrelja , M. Modesti , Appl. Catal., B 2015, 176–177, 225.

[138] M. Sansotera , S. G. Malek Kheyli , A. Baggioli , C. L. Bianchi , M. P. Pedeferri , M. V. Diamanti , W. Navarrini , Chem. Eng. J. 2019, 361, 885.

[139] I. R. Bellobono , R. Stanescu , C. Costache , C. Canevali , F. Morazzoni , R. Scotti , R. Bianchi , E. S. Mangone , G. de Martini , P. M. Tozzi , Int. J. Photoenergy 2006, 2006, 073167.

[140] G. Yang , J. Su , Y. Guo , H. Xu , Q. Ke , J. Mater. Sci. 2017, 52, 5404.

[141] J. Hu , D. Chen , N. Li , Q. Xu , H. Li , J. He , J. Lu , Appl. Catal., B 2017, 217, 224.

[142] Y. Zhao , L. Zhao , J. Han , Y. Xu , S. Wang , Sci. China, Ser. E: Technol. Sci. 2008, 51, 268.

[143] A. Mills , C. Hill , P. K. J. Robertson , J. Photochem. Photobiol., A 2012, 237, 7.

[144] D. Wood , S. Shaw , T. Cawte , E. Shanen , B. Van Heyst , Chem. Eng. J. 2020, 391, 123490.

[145] M. Pelaez , N. T. Nolan , S. C. Pillai , M. K. Seery , P. Falaras , A. G. Kontos , P. S. M. Dunlop , J. W. J. Hamilton , J. A. Byrne , K. O'Shea , M. H. Entezari , D. D. Dionysiou , Appl. Catal., B 2012, 125, 331.

[146] N. Nasrollahi , L. Ghalamchi , V. Vatanpour , A. Khataee , J. Ind. Eng. Chem. 2021, 93, 101.

[147] X. Wang , S. Li , P. Chen , F. Li , X. Hu , T. Hua , Mater. Today Chem. 2022, 23, 100650.

[148] J. Oliveira de Brito Lira , H. G. Riella , N. Padoin , C. Soares , J. Environ. Chem. Eng. 2021, 9, 105068.

[149] C. Maneerat , Y. Hayata , Trans. ASABE 2008, 51, 163.

[150] N. Misdan , W. J. Lau , A. F. Ismail , Desalination 2012, 287, 228.

[151] H. C. Aran , D. Salamon , T. Rijnaarts , G. Mul , M. Wessling , R. G. H. Lammertink , J. Photochem. Photobiol., A 2011, 225, 36.

[152] M. Binazadeh , J. Rasouli , S. Sabbaghi , S. M. Mousavi , S. A. Hashemi , C. W. Lai , Materials 2023, 16, 3526. 37176408 10.3390/ma16093526 PMC10180107

[153] M. Nasir , Juliandri , B. Prihandoko , Procedia Chem. 2015, 16, 184.

[154] M. Safarpour , S. Najjarizad‐Peyvasti , A. Khataee , A. Karimi , J. Environ. Chem. Eng. 2022, 10, 107533.

[155] Z. Kuspanov , B. Bakbolat , A. Baimenov , A. Issadykov , M. Yeleuov , C. Daulbayev , Sci. Total Environ. 2023, 885, 163914. 37149164 10.1016/j.scitotenv.2023.163914

[156] C. H. Kirk , P. Wang , C. Y. D. Chong , Q. Zhao , J. Sun , J. Wang , J. Mater. Sci. Technol. 2024, 183, 152.

[157] F. Amano , K. Tsushiro , Energy Mater. 2024, 4, 400006.

[158] S. Yan , Y. Li , X. Yang , X. Jia , J. Xu , H. Song , Adv. Mater. 2024, 36, 2307967. 10.1002/adma.202307967 37910074

[159] Y. Zhao , Z. Zhou , C. Liu , X. Li , T. Wang , F. Qu , J. Membr. Sci. 2025, 717, 123613.

[160] H. Abdallah , E. S. Mansor , M. Shalaby , A. Shaban , Total Environ. Eng. 2025, 2, 100013.

[161] K. Nelson , A. C. Mecha , H. M. Samuel , Z. A. Suliman , Processes 2025, 13, 163.

[162] J. Zhang , H. Wu , L. Shi , Z. Wu , S. Zhang , S. Wang , H. Sun , Sep. Purif. Technol. 2024, 329, 125225.

[163] T. Wang , Z. Zhou , C. Liu , X. Li , Y. Zhao , J. Membr. Sci. 2025, 717, 123602.

[164] S. Wang , Y. Zhang , X. Zhou , X. Xu , M. Pan , J. Environ. Manage. 2024, 367, 122087. 39111001 10.1016/j.jenvman.2024.122087

[165] B. Abhishek , A. Jayarama , A. S. Rao , S. S. Nagarkar , A. Dutta , S. P. Duttagupta , S. S. Prabhu , R. Pinto , Int. J. Hydrogen Energy 2024, 81, 1442.

[166] S. M. Mousavi , Z. Pouramini , A. Babapoor , M. Binazadeh , V. Rahmanian , A. Gholami , N. Omidfar , R. H. Althomali , W.‐H. Chiang , M. M. Rahman , Chemosphere 2024, 353, 141525. 38395369 10.1016/j.chemosphere.2024.141525

[167] O. Iglesias , M. J. Rivero , A. M. Urtiaga , I. Ortiz , Chem. Eng. J. 2016, 305, 136.

[168] M. N. Chong , B. Jin , C. W. K. Chow , C. Saint , Water Res. 2010, 44, 2997. 20378145 10.1016/j.watres.2010.02.039

[169] H. Ren , P. Koshy , W.‐F. Chen , S. Qi , C. C. Sorrell , J. Hazard. Mater. 2017, 325, 340. 27932035 10.1016/j.jhazmat.2016.08.072

